# Supplementary material for: Koopman global linearization of contact dynamics for robot locomotion and manipulation enables elaborate control
Source: Nat Commun. 2026 Jun 19;17:7749. doi: 10.1038/s41467-026-72485-7 (PMC13434789; doi:10.1038/s41467-026-72485-7)
Supplement: Supplementary file 1 [file 41467_2026_72485_MOESM10_ESM.pdf]

nature portfolio

Peer Review File

Koopman global linearization of contact dynamics for robot locomotion and manipulation enables elaborate control

Corresponding Author: Dr Cormac O'Neill

This file contains all reviewer reports in order by version, followed by all author rebuttals in order by version.

Version 0:

Reviewer comments:

Reviewer #1

(Remarks to the Author)
The paper presents a novel use of the Koopman operator for contact-rich locomotion and manipulation. One of the biggest challenges in robotics is leveraging contact dynamics for planning and control. The paper well-motivates the underlying challenges with contact and posits the Koopman operator as a potential solution. However, the Koopman operator itself has quite a few challenges, mainly that 1) capturing control input to the underlying system is not well defined, and 2) the Koopman operator assumes smoothness in the underlying dynamics.

The proposed approach is to utilize the control-coherent Koopman operator to effectively globally linearize a augmented state system. The paper provides clear conditions for extending the state via the control coherent structure which is quite a notable result and very timely result to include in this paper.

The work applies the control-coherent Koopman operator to systems that undergo contact as it claims; however, it is unclear how the specific contributions are necessarily compatible with the proposed contact-based examples. Specifically, it would be good to overview other, canonical contact-based systems, e.g., a bouncing ball, or the contact-based cart-pendulum problem (see Jin, W., Aydinoglu, A., Halm, M., & Posa, M. (2022, May). Learning linear complementarity systems. In Learning for dynamics and control conference (pp. 1137-1149). PMLR. and Aydinoglu, A., Sieg, P., Preciado, V. M., & Posa, M. (2021). Stabilization of complementarity systems via contact-aware controllers. IEEE Transactions on Robotics, 38(3), 1735-1754.).

Overall, the paper is quite interesting and relevant, but should consider exploring more how the control-coherent Koopman is able to predict contact-based system and under which conditions the contact-type may prove problematic. Below are more specific comments.

Comments:

It would be interesting to see the prediction capabilities of the control-coherent Koopman as the underlying system undergoes more stiff contact dynamics. In particular, does making contact more stiff reduce performance or make the predictions worse over time?

The current examples are roughly quasi-static in terms of the contact model as they are mostly smoothed out or made continuous. It would be interesting to add in an example where the contact dynamics assumptions make it challenging for the Koopman to model, e.g., some kind of linear complementarity constraints.

In the Control-Coherent Kooopman modeling section, expanding on the g_t term and explaining its purpose in that section (near Eq. 8 and 10) would better contextualize its use in the model. In general, it appears that g_t is most likely what drives the benefits of the control-coherent Koopman , making it relevant to include in that section.

In addition, some ablation studies on the choice of g_t, for instance, prediction capabilities as number of basis increases. etc.

Last, the paper could be significantly improved if it compared to the bilinearization control form to deal with Koopman with

control. This has been widely studied and should be compared against.

Reviewer #2

(Remarks to the Author)
This manuscript introduces a novel approach to control contact-rich dynamics in robotics, utilizing Koopman global linearization for Model Predictive Control (MPC) of legged locomotion and dynamic manipulation tasks. The idea of leveraging Koopman operators to create a unified, globally-linear model for systems with switching dynamics due to contact changes is innovative and has the potential to significantly enhance control strategies in robotics. The proposed method, Control-Coherent Koopman (CCK) modeling, demonstrates a promising way to handle the complexities of contact transitions and discontinuities, allowing for real-time MPC implementation.
However, there are several areas where clarification and improvements can be made to enhance the clarity, depth, and applicability of the work. I suggest the following revisions to address these points.
1. While the application of Koopman operators in the control of robotic systems is a central theme of the paper, the manuscript would benefit from a clearer and more intuitive explanation of how the Koopman operator theory specifically applies to systems with multiple types of contact events. The connection between the Koopman operator theory and traditional control methods could be better highlighted, particularly for readers less familiar with the mathematical foundation of Koopman theory. The authors need to include a more detailed comparison of Koopman-based linearization with standard methods such as local linearization and linear complementarity systems, particularly in the context of real-time performance and applicability to non-autonomous systems.
2. The approach of using compliance in the robot-environment interaction to address discontinuities in contact transitions is effective, but further clarification is needed regarding how the compliance values are chosen or tuned in practice. In particular, the role of viscoelasticity in different robot-environment interaction scenarios should be more explicitly discussed.
3. The paper provides robust simulations and hardware experiments, but there could be more emphasis on the limitations of the method in real-world applications, especially in environments with unexpected or variable contact conditions.
4. The numerical results are promising, but some of the key figures, especially those related to the comparison of CCK-MPC against linear-MPC using local linearization, could benefit from clearer labels and additional context. In particular, some of the results could be more thoroughly explained, such as the specific conditions under which CCK-MPC outperforms the other methods.

Reviewer #3

(Remarks to the Author)
This paper presents the results of applying Koopman operators for contact-involved dynamics that often occur in locomotion and manipulation. The assumption of compliant contact is required. The authors claimed that the proposed CCK-MPC method is the first to establish a modeling methodology for subsuming segmented dynamics due to contact changes within a unified, globally-linear, and time-invariant system. With such a linear system, applying real-time MPC becomes available. The proposed method is validated in two specific systems: the rimless wheel locomotion system and the pushing/shoving manipulation system.
I have several comments:
1) Applying Koopman operators for the studied systems may be new, but there has been extensive work on the systems with compliant contacts. For example, in both locomotion and manipulation, there are contact-implicit approaches that can automatically predict contact sequences [1] [2]. I just listed a few here, and the authors can find more studies from the robotics community. For compliant robot-environment interactions, there are various contact models that essentially unify segmented dynamics into one system for planning. To convince people that the method based on Koopman operators outperforms existing contact-implicit approaches, the authors need to provide more fruitful results.
2) Going back to the proposed method, the CCK model is built using simulated data. This constrains the available systems because not all systems can be simulated easily. Assuming a system can be simulated, does the gap between the simulated system and the physical system affect the control performance? How does a user assess the availability and tolerance? Any systematic methods?
3) While generating simulated data, a stabilizing control input with small feedback gains is used. In this case, people may face a chicken-and-egg problem. The goal is to design a real-time MPC control scheme based on the CCK model. However, some control is required in generating data. For simple systems, we can use intuition to inject control efforts. If the system is complex, how can you ensure your controlled data generation is sufficient for determining A matrix?
4) I understand the goal of this paper may be to share a paradigm, so the studied systems are specific and relatively simple/intuitive, and the resulting performance is predictable and not beyond the existing. However, for future users like researchers in the robotics community, we need more generic and deployable approaches. For example, contact-implicit trajectory optimization may suffer from some limitations, but general enough for all contact-involved systems. Researchers can follow it for their own study with improvement and make systems work, which is highly meaningful for robotics. At this moment, the proposed method is only validated on several simple systems, and no further confidence can be provided to other researchers, considering A matrix is determined by simulated data, and most on-going studies involve complex systems like legged manipulators with both locomotion and manipulation in the same system.
5) Going back to the validation provided in this paper, there is no physical validation for the rimless wheel system. Physical experiments are crucial for robotics because implementation problems will arise while deploying your algorithms onto a robot. If the authors do not make this effort, it would be even harder for other researchers who are new to the authors' methodology.
Overall, the exploration in this paper is interesting. However, there are more efforts to be made before it is useful and impactful to the robotics community.
[1] I. Chatzinikolaidis, Y. You and Z. Li, "Contact-Implicit Trajectory Optimization Using an Analytically Solvable Contact

Model for Locomotion on Variable Ground," in IEEE Robotics and Automation Letters, vol. 5, no. 4, pp. 6357-6364, Oct. 2020.
[2] V. Kurtz and H. Lin, "Contact-Implicit Trajectory Optimization with Hydroelastic Contact and iLQR," 2022 IEEE/RSJ International Conference on Intelligent Robots and Systems (IROS), Kyoto, Japan, 2022, pp. 8829-8834.

Version 1:

Reviewer comments:

Reviewer #1

(Remarks to the Author)
The authors provided a substantive rebuttal with ample examples that directly respond to my comments. I look forward to the published version of the paper with the provided example.

(Remarks on code availability)

Reviewer #2

(Remarks to the Author)
The paper has been improved significantly. I just have one more suggestion.

The comparison with bilinear MPC is great. It would also be very convincing to compare your method's performance and speed against a more standard approach, like an MPC that uses local linearization along a pre-planned trajectory (LTV-MPC).

(Remarks on code availability)

Reviewer #3

(Remarks to the Author)
Thanks for your reply. I have the following comments:

1) My original concern was not about runtime but about modeling capability and relevance. The authors' reply focuses mainly on computational speed, which does not address whether the proposed Koopman-based approach actually improves the modeling of compliant or contact-rich dynamics compared with the extensive literature on contact-implicit and compliant-contact methods. Moreover, the presented results are limited to simple, low-dimensional systems, which makes the claimed speed advantage less compelling to a broader robotics audience concerned with scalability to manipulation, locomotion, or whole-body control. Without evidence that CCK maintains accuracy or generality on more realistic multi-contact tasks, or comparative evaluations against established contact-implicit frameworks, it remains difficult to assess whether the proposed method offers meaningful advantages beyond faster inference on toy examples.

2) While I appreciate the authors' expanded discussion, the response still does not fully address my underlying concern. My question was not simply about whether data-driven methods can be used, but about the practical availability and reliability of constructing CCK models for real systems that cannot be easily simulated or modeled. The reply lists candidate data-driven Koopman techniques and acknowledges that sim-to-real gaps remain largely empirical, but it does not provide clear guidance on how a user should assess when a system satisfies the CCK compatibility conditions, how much sim-to-real discrepancy can be tolerated before control performance degrades, or whether any systematic diagnostic methods exist for evaluating model adequacy. Therefore, while the response provides useful context, it does not fully answer the original question regarding the practical feasibility and robustness of constructing CCK models for systems that are not straightforward to simulate or whose dynamics contain significant uncertainty.

4) Thank you for the detailed clarification, but my concern is not about the theoretical rigor of the CCK formulation, but about its practical generality and deployability for future users in the robotics community. The mathematical conditions for CCK applicability are helpful, but they do not substitute for evidence that the approach can scale to the kinds of high-dimensional, hybrid, contact-rich systems that are common in robotics research. At present, the method has only been demonstrated on relatively simple and low-dimensional examples; thus, it remains difficult for users to judge whether CCK-MPC will behave reliably, remain tractable, or maintain modeling fidelity on more complex platforms. Contact-implicit trajectory optimization, despite its limitations, can be used because it is general enough for arbitrary contact-involved systems and provides a deployable pipeline for diverse tasks. Without broader validation or benchmarks on more challenging real-world systems, it is hard to conclude that CCK-MPC offers general advantages or can serve as a practical tool beyond the specific examples shown.

In summary, while the CCK formulation is theoretically elegant, the authors' responses do not fully resolve concerns about its practical relevance, scalability, and usability for real-world robotic systems. The current validation is confined to simple, low-dimensional examples, and the replies emphasize computational speed and theoretical generality without providing

concrete evidence that CCK can robustly model or control the complex and contact-rich dynamics encountered in modern robotics. Moreover, questions regarding how users should assess applicability, manage sim-to-real discrepancies, and reliably construct CCK models for systems that are difficult to simulate remain insufficiently addressed. As a result, it is still unclear whether the proposed approach can serve as a broadly deployable tool for the robotics community.

(Remarks on code availability)

Version 2:

Reviewer comments:

Reviewer #3

(Remarks to the Author)
Thanks very much for your detailed response. My concerns have been fully addressed.

(Remarks on code availability)

Open Access This Peer Review File is licensed under a Creative Commons Attribution 4.0 International License, which permits use, sharing, adaptation, distribution and reproduction in any medium or format, as long as you give appropriate credit to the original author(s) and the source, provide a link to the Creative Commons license, and indicate if changes were made.
In cases where reviewers are anonymous, credit should be given to 'Anonymous Referee' and the source.
The images or other third party material in this Peer Review File are included in the article’s Creative Commons license, unless indicated otherwise in a credit line to the material. If material is not included in the article’s Creative Commons license and your intended use is not permitted by statutory regulation or exceeds the permitted use, you will need to obtain permission directly from the copyright holder.
To view a copy of this license, visit https://creativecommons.org/licenses/by/4.0/

Point-by-Point Response to the Reviewers' Comments

NCOMMS-25-48772

Koopman Global Linearization of Contact-Rich Dynamics Enables Robots to Discover Elaborate Control for Locomotion and Manipulation

The original title above exceeded the word limit: 17 words > 15 words limit. Therefore, the title is slightly modified to:

Koopman Global Linearization of Contact Dynamics for Robot Locomotion and Manipulation Enables Elaborate Control

All the reviewers' comments are reproduced verbatim and are shown below in *Italic*, while the authors' response is written in a regular font.

Each point raised by the reviewers is labeled as ***R**x-**P**y*, meaning that Reviewer *x*, Point *y*.

Reviewer #1 (Remarks to the Author):

The paper presents a novel use of the Koopman operator for contact-rich locomotion and manipulation. One of the biggest challenges in robotics is leveraging contact dynamics for planning and control. The paper well-motivates the underlying challenges with contact and posits the Koopman operator as a potential solution. However, the Koopman operator itself has quite a few challenges, mainly that 1) capturing control input to the underlying system is not well defined, and 2) the Koopman operator assumes smoothness in the underlying dynamics.

The proposed approach is to utilize the control-coherent Koopman operator to effectively globally linearize a augmented state system. The paper provides clear conditions for extending the state via the control coherent structure which is quite a notable result and very timely result to include in this paper.

R1-P1 *The work applies the control-coherent Koopman operator to systems that undergo contact as it claims; however, it is unclear how the specific contributions are necessarily compatible with the proposed contact-based examples. Specifically, it would be good to overview other, canonical contact-based systems, e.g., a bouncing ball, or the contact-based cart-pendulum problem (see Jin, W., Aydinoglu, A., Halm, M., & Posa, M. (2022, May). Learning linear complementarity systems. In Learning for dynamics and control conference (pp. 1137-1149). PMLR. and Aydinoglu, A., Sieg, P., Preciado, V. M., & Posa, M. (2021). Stabilization of complementarity systems via contact-aware controllers. IEEE Transactions on Robotics, 38(3), 1735-1754.).*

Response to *R1-P1*

We thank the reviewer for their insightful comments and appreciate their point on the importance of canonical contact-based examples for ease-of-communication within our field.

The authors agree to address the applicability to the suggested canonical tasks. In past-work, however, the authors did apply Koopman modeling to the suggested canonical problems.

1. **The bouncing ball**

The bouncing ball is the simplest example of contact-based dynamical systems we have investigated for verifying the global linearization method based on the Koopman operator theory. As shown below, we can see that the globally linear model with 100 observables can perform reasonably well when making predictions across impacts. Even after two bounces, the Koopman model can predict the ball motion well. The bouncing ball example was recently included in one of the author's dissertations [51], which is now cited in the main manuscript. The result below is a reproduction, Fig.1.

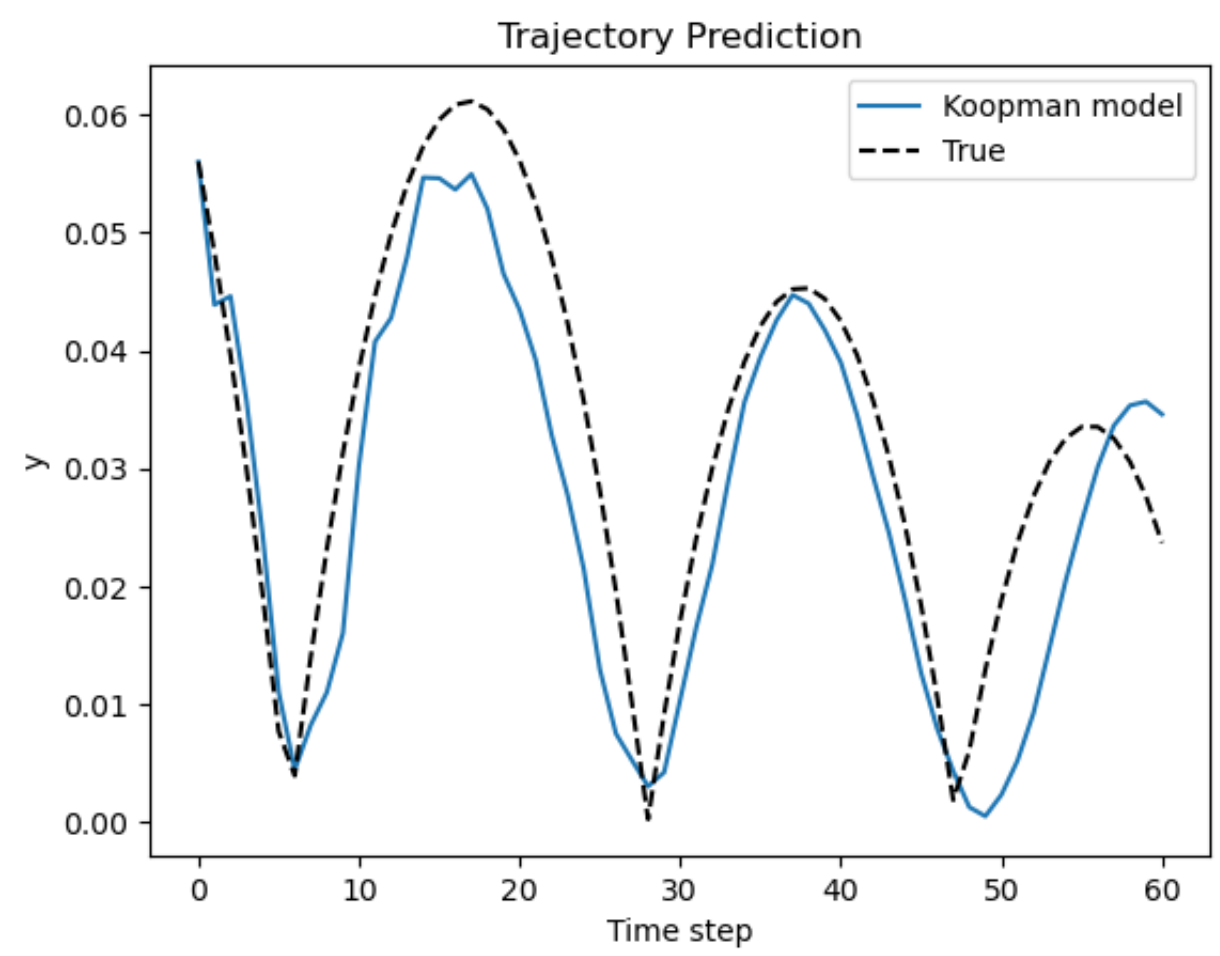

PTP Fig. 1. Koopman modeling of a ball bouncing on a floor

2. **Cable-suspended balls**: In addition to the bouncing ball canonical task, the authors' group has applied Koopman operators to a cable suspended ball, which is similar to the bouncing ball dynamics. A ball suspended with an elastic cable is similar to an elastic ball bouncing on a floor. It is a switched dynamical system due to transition between taut and slack of the cable. When the ball is suspended with two cables, its dynamics become more complex. Nonetheless, we have obtained an excellent prediction with use of a large number of Radial Basis Functions as observables. See PTP Figs. 2 and 3 below. The work has been published in [26].

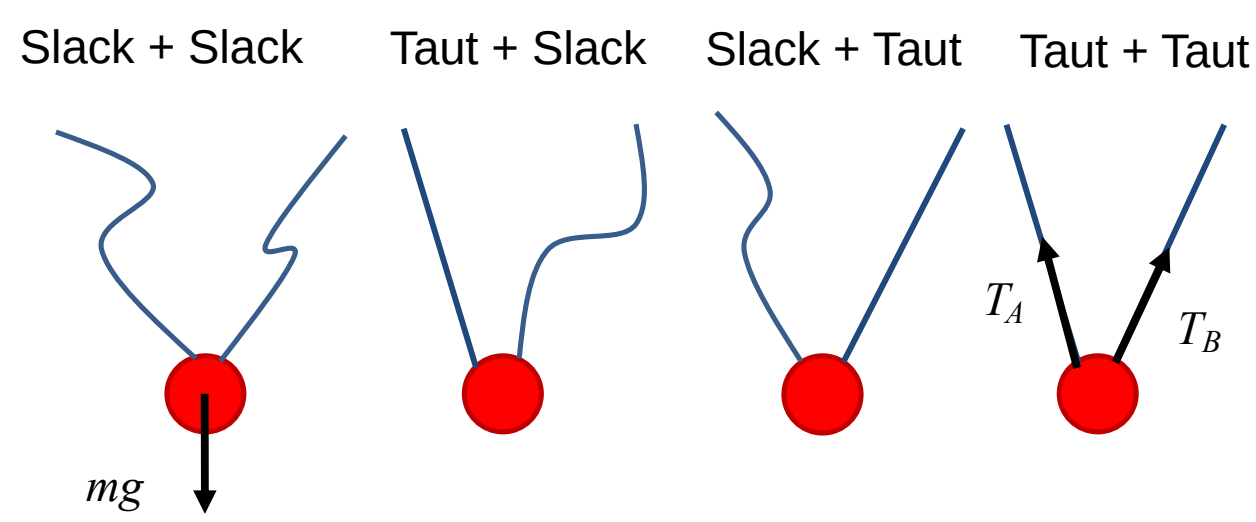

PTP Fig.2 Taut-slack states of a ball suspended with two cables

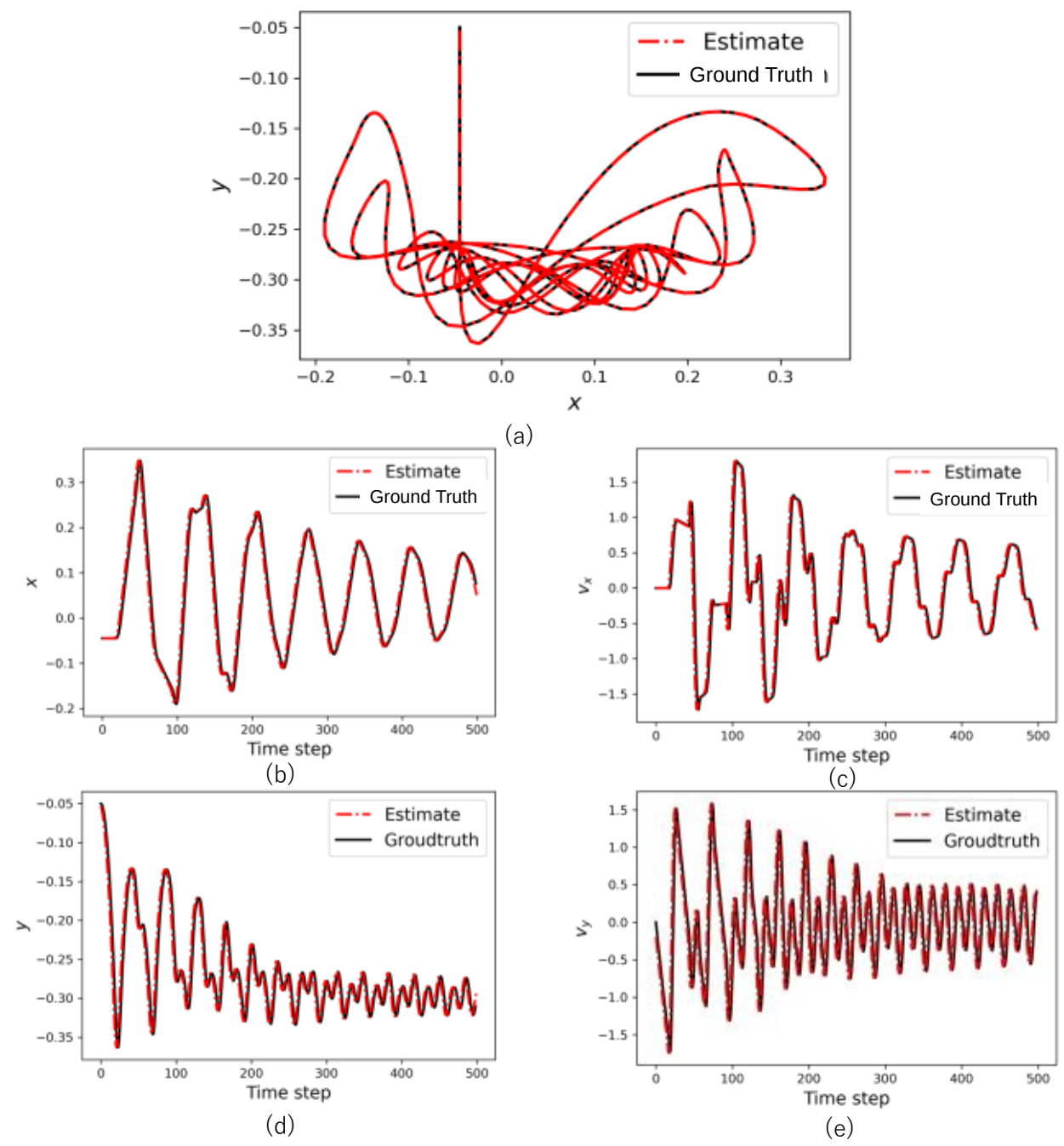

PTP Fig.3 Estimated ball motion with a high-order Koopman model

Please note that the above bouncing ball tasks are autonomous systems with no control input. The cart-pendulum problem is a non-autonomous system with control, to which CCK-MPC is applicable.

3. **A wall – bound cart-pole** We have conducted a simulation experiment by applying our CCK-MPC to the same system described in the suggested reference. As shown in Supplementary Materials, the CCK-MPC has achieved more than "comparable" results. This is to clarify the reviewer's concern. We would like to emphasize, however, that the CCK MPC has achieved much more than those tasks that the traditional methods have achieved.

To show the ability for Koopman models to control contact-based systems, we generate a simple Koopman LQR controller for the cart-pole system with compliant walls. This model, described in Aydinoglu et al., which was suggested by the reviewer, is given by a simple cart-pole with two generalized coordinates ($\boldsymbol{\theta} = [x_c, \theta_p]$) and compliant walls that are centered a

distance d from the origin. Here, the contact dynamics are a simple linear spring, whose contact force is given by :

$$0 \leq \lambda_1 \perp l \sin(\theta_p) - x_c + \frac{1}{k} \lambda_1 + d \geq 0$$

$$0 \leq \lambda_2 \perp x_c - l \sin(\theta_p) + \frac{1}{k} \lambda_1 + d \geq 0$$

Where λ_1 and λ_2 correspond to the right and left wall reaction forces respectively. In this case, if we use the small angle assumption ($\sin(\theta_p) \approx \theta_p$), the contact forces can be described using linear complementarity constraints that yield a single solution and continuous dynamics.

We consider a case where the distance between the two walls, $2d$, is small, which is more challenging because the pole contacts the walls and bounces back more frequently. This also makes the angle θ_p deviation from the unstable equilibrium small, so the control input is linear affine in the equations of motion. Thus, we are able to obtain the Koopman B matrix analytically from the equations of motion. However, a Koopman model can be constructed that takes into account the state dependent nature of the control input using the Control Coherent Koopman method, either using the actuator pre-filter method [PTP-1] or modeling the actuator powertrain dynamics [35].

The equations of motion for the cart-pole system with compliant walls is given below:

$$\ddot{\boldsymbol{\theta}} = \mathrm{H}^{-1}(\boldsymbol{\theta})\left[\mathrm{C}(\boldsymbol{\theta}, \dot{\boldsymbol{\theta}}) + \mathrm{G}(\boldsymbol{\theta}) + \boldsymbol{u}\right] + \mathrm{J}_c(\boldsymbol{\theta})\, \boldsymbol{\Lambda}$$

$$\mathrm{H}(\boldsymbol{\theta}) = \begin{bmatrix} m_c + m_p & -l\, m_p \cos(\theta_p) \\ -l m_p \cos(\theta_p) & l^2 m_p \end{bmatrix}$$

$$\mathrm{C}(\boldsymbol{\theta}, \dot{\boldsymbol{\theta}}) = \begin{bmatrix} \dot{\theta}_p^2\, l m_p \sin(\theta_p) \\ 0 \end{bmatrix}$$

$$\mathrm{G}(\boldsymbol{\theta}) = \begin{bmatrix} 0 \\ -g\, l m_p \sin(\theta_p) \end{bmatrix}$$

$$\boldsymbol{u} = \begin{bmatrix} u \\ 0 \end{bmatrix}$$

$$\mathrm{J_C}(\boldsymbol{\theta})\boldsymbol{\Lambda} = \begin{bmatrix} 1 & -l\cos(\theta_p) \\ 0 & -l\sin(\theta_p) \end{bmatrix} \begin{bmatrix} -\lambda_1 + \lambda_2 \\ 0 \end{bmatrix}$$

To obtain an analytical B matrix for the Koopman model, we use the linear control input term:

$$\widetilde{\mathrm{B}}\, u = \begin{bmatrix} \frac{1}{m_c} \\ \frac{1}{l\, m_c} \end{bmatrix} u$$

The model uses 25 radial basis function observables placed with K-means++. The simulation and Koopman time step were both 0.01 seconds, and the dynamics were integrated using forward Euler.

In PTP Fig. 4, we show the results of the Koopman LQR model compared to a typical cart-pole LQR controller with no knowledge of the walls. The same state and control weights were used between the two controllers. The regular LQR controller rebounds against the walls and is unable to stabilize, while the Koopman LQR controller hits the right wall but is able to bring the cartpole to the origin. For 1000, six-second simulations, 79.9% of the simulations using the KLQR controller had an average MSE in the last second below 0.01, but only 10.7% of simulations below 0.001. Contrast this with the regular LQR controller, who for the same initial conditions was able to get 43.7% of simulations below 0.01 and 42.8% below 0.001. While the Koopman LQR controller was more consistent in stabilizing the cart-pole, small errors in the model keeps the cart-pole from arriving at the unstable equilibrium. Regular LQR can bring the cart-pole to this unstable equilibrium, but only if the model is not greatly impacted by the wall collisions. The initial conditions for these simulations were uniformly sampled from the following ranges:

$$|x_c| \leq d, |\dot{x_c}| \leq 1.5$$

$$\theta_p = 0, |\dot{\theta_p}| \leq 1$$

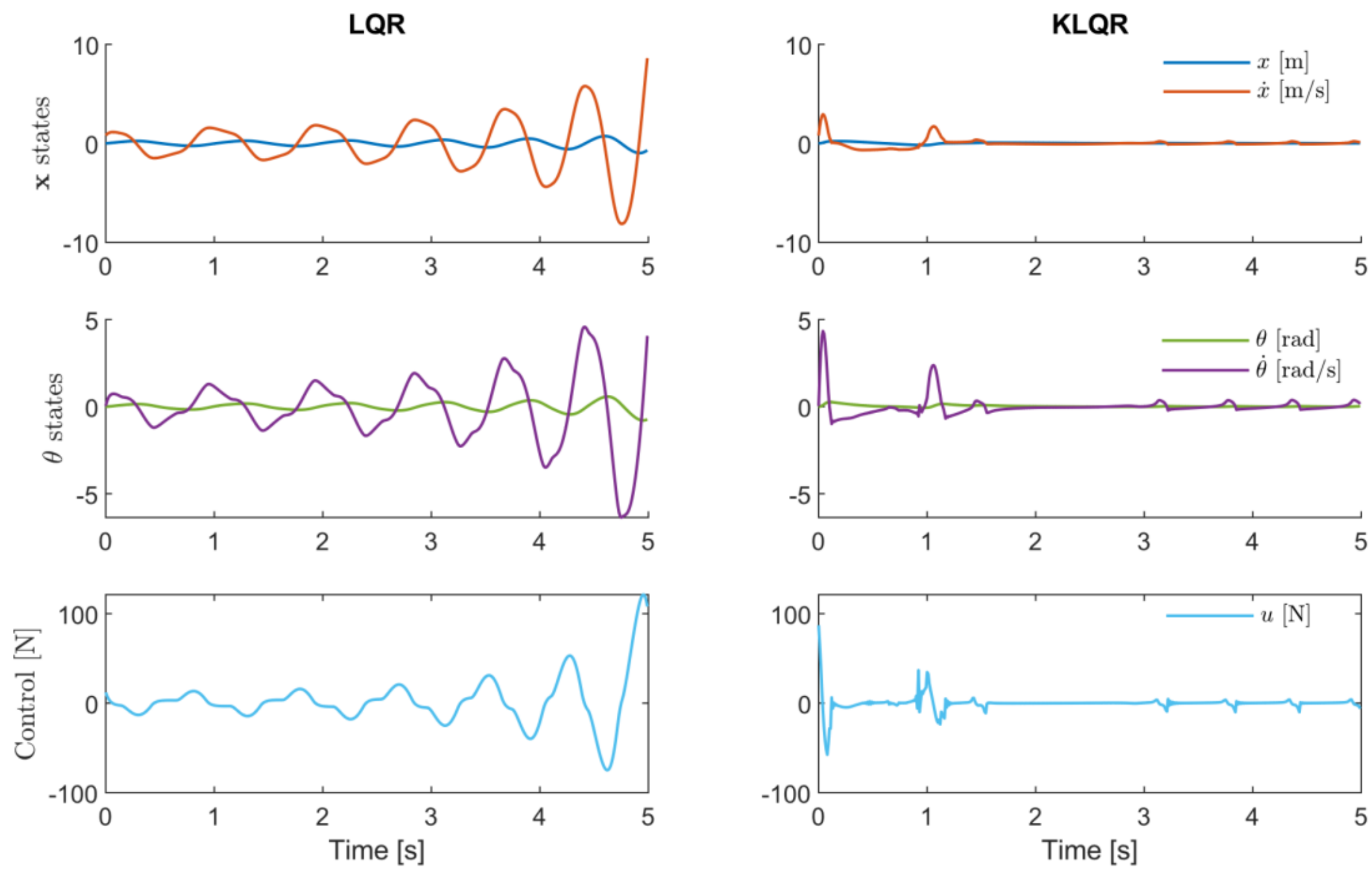

PTP Fig.4. Comparison of Koopman LQR vs LQR for cart-pole with walls
Shown for a single trajectory. KLQR, which has knowledge of the wall location and dynamics, is capable of stabilizing the cart-pole, while the LQR controller bounces off the walls repeatedly. Please see Supplementary Movie 7 for the animation of these two trajectories.

These results fully demonstrate that Koopman global linearization is applicable to the canonical problems of both autonomous and non-autonomous systems. Furthermore, the cart-pole interacting with a pair of walls is a good canonical example. We have included the cart-pole example as a supplementary material, Supplementary Discussion 1.3, and mentioned it in the manuscript before presenting the rimless wheel and dynamic pushing systems, which are more complex.

Overall, the paper is quite interesting and relevant, but should consider exploring more how the control-coherent Koopman is able to predict contact-based system and under which conditions the contact-type may prove problematic. Below are more specific comments.

Thank you for your support and suggestion. The reviewer's concerns are fully addressed below in response to specific issues.

Comments:

R1-P2 *It would be interesting to see the prediction capabilities of the control-coherent Koopman as the underlying system undergoes more stiff contact dynamics. In particular, does making contact more stiff reduce performance or make the predictions worse over time?*

The authors appreciate this question, which is the key to the applicability of CCK. We had a paragraph discussing the stiffness issue in the original manuscript, but it must be extended. Also,

this question is aligned with the second point of Reviewer 2, R2-P2. Please refer to the response to R2-P2 below for more detail.

We have investigated the effect of contact stiffness upon the Koopman modeling accuracy, control bandwidth, and overall control performance. We have found that a high stiffness slows down the convergence speed of Koopman operators, needing a larger number of observables yet reducing the prediction accuracy. There is a lower limit to the contact stiffness that can produce reliable prediction and MPC control performance. See PTP Figs. 5 and 6.

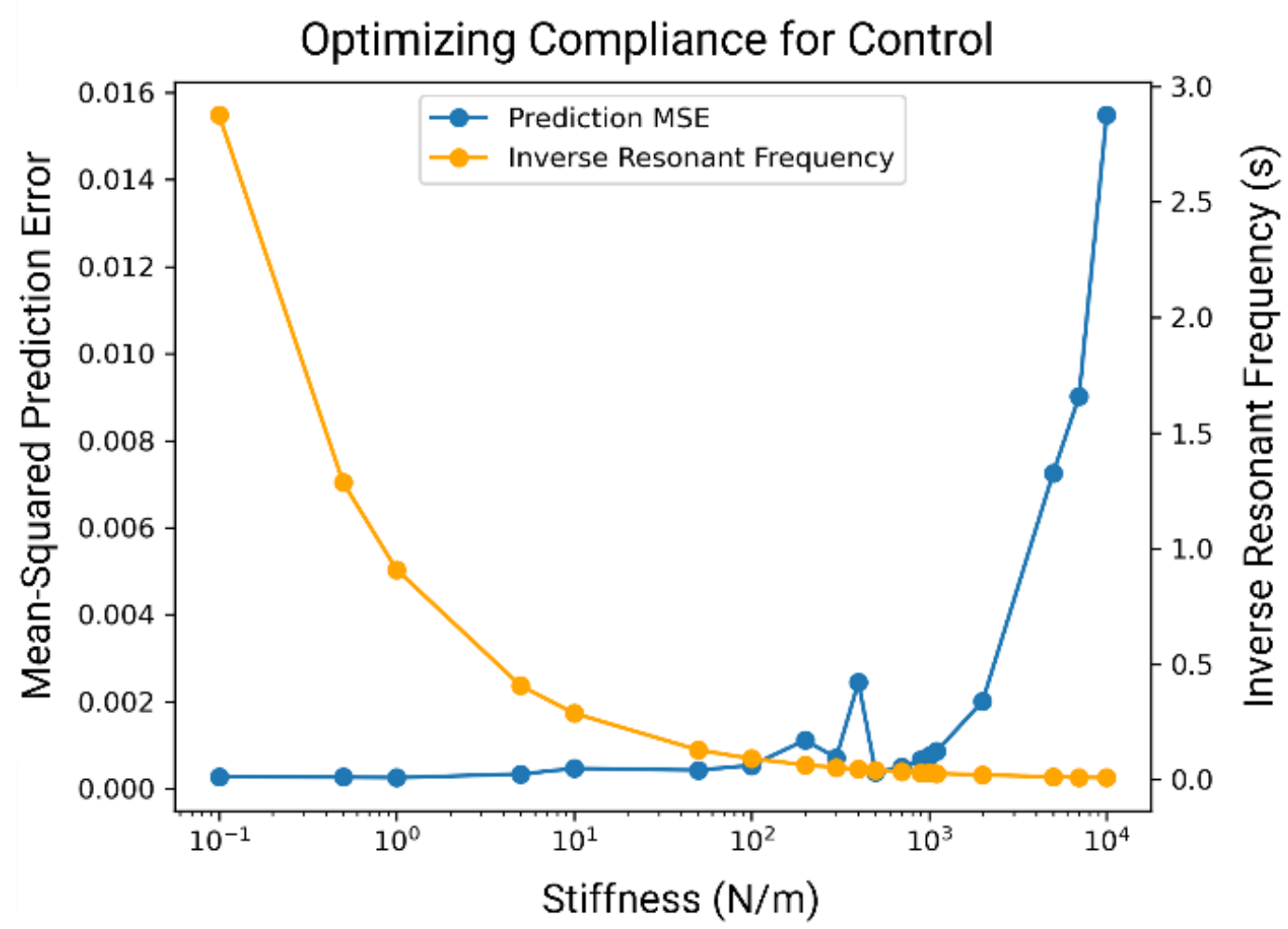

PTP Fig.5 Mean-squared prediction error v.s. contact stiffness (blue) and the inverse resonance frequency of mass-spring system as a surrogate of control bandwidth

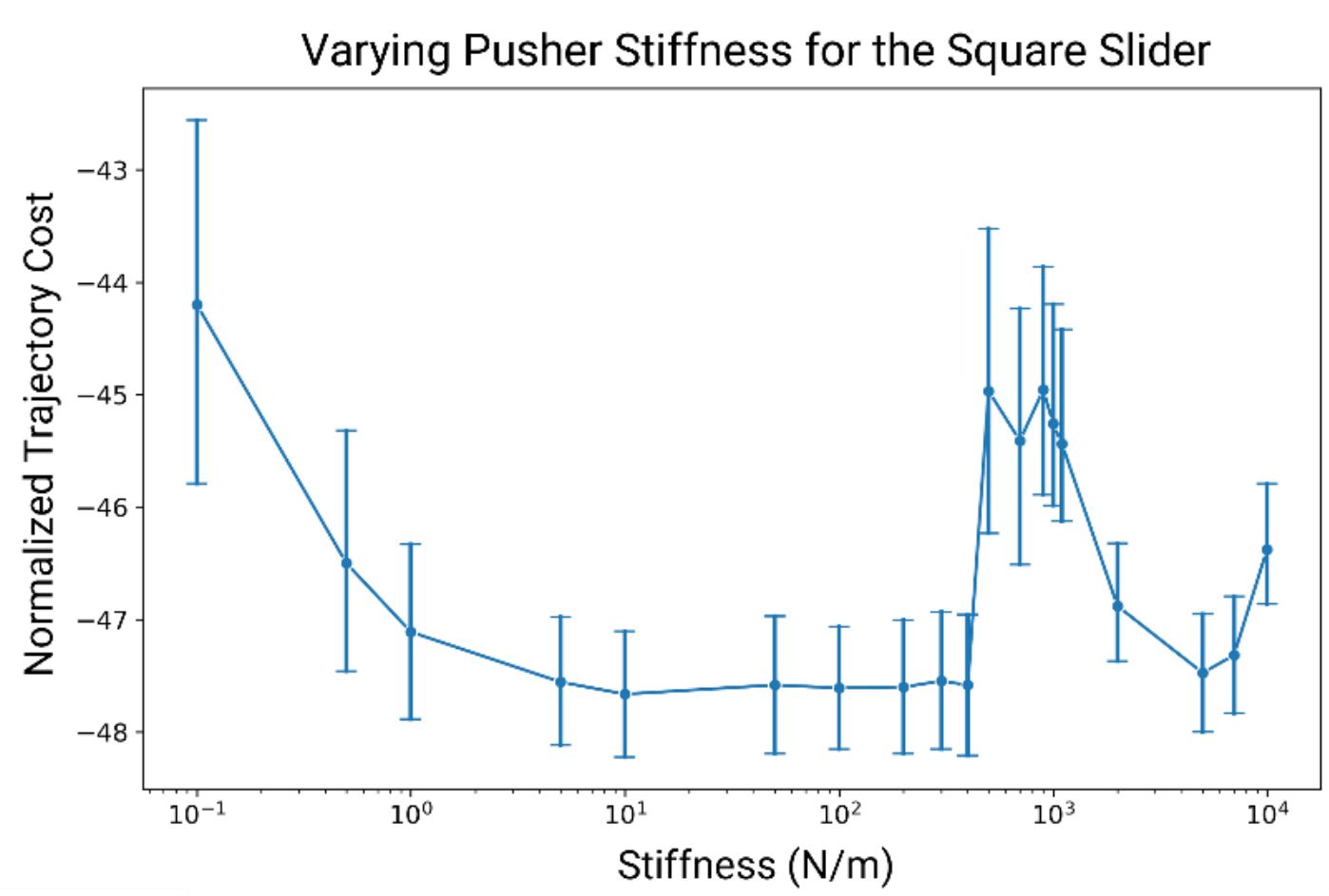

PTP Fig.6 MPC control performance in terms of the optimization index cost v.s. contact stiffness

If the pusher stiffness is too low, on the other hand, the control bandwidth decreases and the control authority deteriorates. These two conflicting issues suggest that there is an acceptable range of stiffness that the CCK-MPC produces fruitful results. An interesting view of this contact stiffness is that we can treat the contact stiffness as a "design parameter" that we should tune to improve overall control performance. In the pushing and shoving manipulation, the pusher stiffness can be selected to robustly improve the performance. Making a trade-off between the two conflicting requirements, we can find an optimal stiffness range for which high control

authority and model prediction accuracy are guaranteed. The pusher can also contact an object for a longer time, as opposed to instantaneous contact, and thereby increase controllability in a broader sense. CCK-MPC provides an effective and comprehensive framework for designing the stiffness range.

We have extended the Discussion section by addressing this contact stiffness issue in the fourth and fifth paragraphs. We have also added more details about the numerical analysis of the effect of contact stiffness in Supplementary Discussion 1.2.

R1-P3 *The current examples are roughly quasi-static in terms of the contact model as they are mostly smoothed out or made continuous. It would be interesting to add in an example where the contact dynamics assumptions make it challenging for the Koopman to model, e.g., some kind of linear complementarity constraints.*

Response to *R1-P3*

We thank the reviewer for this insightful comment, and we apologize if we are misunderstanding their meaning. To the best of our knowledge, whether or not the contact model is continuous in a C^0 sense is unrelated to whether it is dynamic. Compliant contact can represent dynamic contact when inertial forces are significant, and when friction is not assumed to be excessively large. This contrasts with quasi-static and quasi-dynamic models that assume inertial forces are negligible and all other forces are balanced in every individual time slice.

In the current work, we specifically avoid making the quasi-static and quasi-dynamic assumptions. The ability of the CCK-MPC to provide dynamic control for the planar pushing system and rimless wheel, while still allowing for reactive control rates, is a key contribution. Linear complementarity methods can also avoid the quasi-static and quasi-dynamic assumptions, but have not yet been able to achieve real-time rates (defined in this work as faster than 100Hz) without using parallelization via ADMM methods, local linearization, and/or domain-specific guidance for the location of the robot end-effector in the pushing case. That being said, we do not wish to claim that the proposed CCK-MPC approach is inherently better (or otherwise) than LCC methods. LCC approaches have demonstrated some capabilities with additional tools and assumptions. In this work, we seek to present CCK-MPC as an alternative tool in the robotic engineer's toolkit: a way to approach contact-rich control via a completely <u>convex MPC formulation</u>. We do not doubt that there will be cases in which one method will be better-suited to the other. Continuous modeling with some form of compliance has been used broadly because of its compelling features. The limitation to the Koopman operator approach due to stiffness has been addressed in the previous question.

We have also considered another interpretation of the reviewer's comment. If the reviewer refers to a "dynamic" contact model possessing additional state variables, which has been investigated in system dynamics, applied mechanics, and tribology fields, the proposed

Koopman approach can effectively handle complex nonlinearities involved in such higher-order nonlinear contact models.

R1-P4 *In the Control-Coherent Kooopman modeling section, expanding on the g_t term and explaining its purpose in that section (near Eq. 8 and 10) would better contextualize its use in the model. In general, it appears that g_t is most likely what drives the benefits of the control-coherent Koopman , making it relevant to include in that section.*

Response to *R1-P4*

Thank you for pointing out the mathematical presentation issue. The authors agree with the reviewer on three major points. 1) Page 6 only presented mathematical conditions and derivation of CCK formulation, rather than explaining why it was formulated in that way, the trick of CCK representation. 2) The notation was confusing since all the observables were represented as $g_i(\mathbf{x})_{i=1}^{\infty}$, while the first *m* observables were actuator state variables and the rest of the part was denoted by $\mathbf{g}_t = (g_{m+1},\ g_{m+2},\cdots)^T$. 3) The key point of CCK formulation was not completed in the Results section. It was divided between the Results section and the Methods section, which made it difficult to follow. Furthermore, the mathematical proof of CCK representation – why the class of non-autonomous systems satisfying the conditions can be represented as a Linear Time-Invariant (LTI) system - was not fully addressed. This might have confused the reviewer.

To improve the manuscript on these shortcomings, the following revision and addition have been made:

a) A brief, intuitive introduction to Koopman operator theory has been included to clarify the basic concept of observables – Supplementary Discussion 1.1.
b) The second half of the section "Control-Coherent Koopman Modeling" in the Results section has been rewritten to better explain the key formula of CCK modeling. Specifically, i) the notation of **z**, **p**, and **g** as well as the variables of the associated autonomous system have been clarified. ii) The method for incorporating the linear control term $\boldsymbol{B}_p\boldsymbol{u}_t$ into the autonomous system's Koopman operator **A** has been explained by constructing the state transition equation of $\boldsymbol{p}_t$. It has also been mentioned that the actuator state variables are placed at the first *m* variables in the observables vector $\boldsymbol{z}_t$ in order to facilitate the integration of $\boldsymbol{B}_p\boldsymbol{u}_t$ to the autonomous system. This allows us to use the actuator part of the state equation (4) in the state equation in the lifted space. iii) The distinction between the actuator state at t+1 in the non-autonomous system, $\boldsymbol{p}_{t+1}$, and that of the autonomous system, $\overline{\boldsymbol{p}}_{t+1}$, has been clarified.
c) The time evolution of embeddings $\mathbf{g}_t = (g_{m+1},\ g_{m+2},\cdots)^T$ was not accurately addressed in the original manuscript. The transition of these observables from $\boldsymbol{g}_t$ to $\boldsymbol{g}_{t+1}$ is more involved because the autonomous system's Koopman operator **A** generates only $\overline{\boldsymbol{g}}_{t+1}(\overline{\boldsymbol{p}}_{t+1}, \boldsymbol{q}_{t+1})$ and, therefore, the conversion from $\overline{\boldsymbol{g}}_{t+1}(\overline{\boldsymbol{p}}_{t+1}, \boldsymbol{q}_{t+1})$ to $\boldsymbol{g}_{t+1}(\boldsymbol{p}_{t+1}, \boldsymbol{q}_{t+1})$ is necessary. This additional conversion can be made by adding a linear control term $\boldsymbol{B}_g\boldsymbol{u}_t$ to the time evolution equation of $\boldsymbol{g}_t$. Although this conversion is added, the total state transition is still linear and time invariant, as shown in Equation (11) in the revised manuscript. The computation of the gain matrix $\boldsymbol{B}_g$ is more technical and involved. As such, the derivation has been presented in the Methods section. This is the complete proof of the CCK formulation and is a new contribution of the current work.

R1-P5 *In addition, some ablation studies on the choice of g_t, for instance, prediction capabilities as number of basis increases. etc.*

Response to *R1-P5*

Ablation or truncation of infinite observables to a finite and manageable order is an important and classical problem of Koopman operators. The authors' group has been conducting extensive studies on the effect of ablation / truncation. These include [5, 26, 56]. Many other papers, too, have addressed the truncation issue. As for the current work, the doctoral dissertation of an author addresses the truncation of various types of observable functions. The Discussion section of the current work briefly mentions the order of observables and more details are presented in Supplementary Discussion 1.5.

R1-P6 *Last, the paper could be significantly improved if it compared to the bilinearization control form to deal with Koopman with control. This has been widely studied and should be compared against.*

Response to *R1-P6*

We have compared CCK-MPC to the bilinearization methods by conducting a simulation experiment. The bilinear Koopman MPC achieves high prediction accuracy but slightly underperformed in MPC control performance. Most notably, however, the computation time is significantly different; the bilinear MPC took more than 50 times longer than the proposed CCK in MPC computation. The bilinear MPC is not convex optimization and is difficult to apply to real-time, reactive control. The 7th paragraph of the Discussion section addresses this and more details of the computation and comparison are presented in Supplementary Discussion 1.4.

Reviewer #2 (Remarks to the Author):

This manuscript introduces a novel approach to control contact-rich dynamics in robotics, utilizing Koopman global linearization for Model Predictive Control (MPC) of legged locomotion and dynamic manipulation tasks. The idea of leveraging Koopman operators to create a unified, globally-linear model for systems with switching dynamics due to contact changes is innovative and has the potential to significantly enhance control strategies in robotics. The proposed method, Control-Coherent Koopman (CCK) modeling, demonstrates a promising way to handle the complexities of contact transitions and discontinuities, allowing for real-time MPC implementation.

Thank you for the support.

However, there are several areas where clarification and improvements can be made to enhance the clarity, depth, and applicability of the work. I suggest the following revisions to address these points.

R2-P1 *While the application of Koopman operators in the control of robotic systems is a central theme of the paper, the manuscript would benefit from a clearer and more intuitive explanation of how the Koopman operator theory specifically applies to systems with multiple types of contact events. The connection between the Koopman operator theory and traditional control methods could be better highlighted, particularly for readers less familiar with the mathematical foundation of Koopman theory. The authors need to include a more detailed comparison of Koopman-based linearization with standard methods such as local linearization and linear complementarity systems, particularly in the context of real-time performance and applicability to non-autonomous systems.*

Response to *R2-P1*

The authors agree that the Koopman operator theory should be explained more intuitively to the broad Nature Communications readership. To demystify the Koopman lifting linearization, a brief and intuitive tutorial has been added to the supplementary information, Supplementary Discussion 1.1, the material of which is based on an author's graduate course at MIT. As long as the reader has an undergraduate-level function analysis background, e.g. Fourier Series Expansion, they will gain an intuitive understanding how a nonlinear dynamical system can be converted to a globally linear, time-invariant system in a lifted space.

Following the reviewer's comments, we have revised the Results section to better explain the difference between the Koopman global linearization and the standard local linearization. Specifically, the three paragraphs explaining Figures 3 and 4 of the rimless wheel, pages 10-11, focus on the difference of the global and local linearization and their impact upon model predictive control.

To clarify the reviewer's question "*how the Koopman operator theory specifically applies to systems with multiple types of contact events*", a new equation (12) has been added to the end of the CCK modeling section, manifesting the "segmented" dynamical systems. As long as the segmented equations are continuous at the boundaries and satisfy other conditions, a segmented dynamical system can be converted to a globally linear system. This point was not manifested in the original manuscript, but is clearly stated at the end of the CCK modeling section.

The connection between the Koopman operator theory and traditional control methods in general is a broad topic. However, the relationship between the two can be briefly explained with regard to MPC and optimal control. At least the following two are important background knowledge:

- As the number of state variables increases, CCK lifted dynamical systems are no longer controllable. However, MPC and optimal control do not require controllability. Therefore, the lack of controllability does not invalidate the use of Koopman models for MPC.
- The major advantage of the use of CCK for optimal control is that the globally linear CCK model allows for convex optimization, although the original system is nonlinear when represented with independent state variables. Convex optimization does not incur local minima problems, and its computation is an order-of-magnitude simpler and faster. It is advantageous for real-time control.

These properties that must be understood to correctly apply Koopman models to control design are explained at the end of the subsection, "MPC in Lifted Space" in the Methods section.

We hope that these additional explanations and background information will enhance the readability of the paper.

R2-P2 *The approach of using compliance in the robot-environment interaction to address discontinuities in contact transitions is effective, but further clarification is needed regarding how the compliance values are chosen or tuned in practice. In particular, the role of viscoelasticity in different robot-environment interaction scenarios should be more explicitly discussed.*

Response to *R2-P2*

This is a critical point. The authors appreciate the reviewer's comment. This comment / question pertains to the concern of Reviewer 1, ***R1-P2***. As mentioned in the response to ***R1-P2***, we have investigated the effect of contact stiffness upon the accuracy of Koopman model, control bandwidth and authority, and overall control performance. Reviewer 2 further asking what if the robot interacts with different environments. We first address the effect of contact stiffness upon the prediction accuracy of a CCK model, control bandwidth, and overall MPC performance, although largely overlapped with the response to ***R1-P2***.

We have observed the following properties as the contact stiffness increases:

1) Worse Prediction Accuracy: When trying to track a controlled trajectory, CCK (in general) performs worse when approximating systems with extremely large stiffness values. Modeling a stiff contact with a Koopman approach requires a large number of observables for accurate prediction, while it is difficult to make a sweeping generalization for all systems and all observable functions. Furthermore, stiff contact model requires a higher sampling rate for discrete-time computation. As such, a larger stiffness is **worse** for performance.
2) Responsiveness to Control Inputs: as compliance is introduced to the system, there is an increasing phase lag between the control inputs moving the pusher and the resulting motion of the slider. This reduced control authority negatively impacts the performance of the controller pusher-slider system since more aggressive control inputs would be required to exert a given force on the slider. Moreover, short-horizon controllers - such as the Koopman MPC approach described in this work - could be more adversely impacted since the downstream effects of a given control input on the slider would be less visible to the predictive controller. As such, a larger stiffness is **better** for performance.

We can see the impact of these two influences by comparing the prediction of CCK for a controlled trajectory, and the "inverse resonant frequency" (used as a proxy for the responsiveness of the system – we separately found that this closely tracked the velocity that could be imparted onto the slider for a fixed control input with different stiffness values). PTP Fig.5, shown previously in response to ***R1-P2***, plots these two factors against the contact stiffness for the planer pushing manipulation task. These two factors are conflicting to each other. Both extremely high and low stiffness ranges are not usable.

Performing a range of simulations for the square slider with varying stiffness values, we can find the overall MPC control performance. As shown in PTP Fig.6, the stiffness range between 1 N/m to 400 N/m gives an excellent trajectory tracking cost.

This detailed engineering analysis has been presented in the doctoral thesis of an author [51], where all the conditions and assumptions for numerical simulations are provided. The thesis has been cited as a reference.

Based on these results, we can make the following discussion points.

1. The proposed CCK-MPC method works properly for a range of contact stiffness excluding extremely low and high stiffnesses. For the pushing manipulation task, this range covers 1 N/m to 400 N/m.
2. The contact stiffness is determined in part by the design of the pusher in the case of the pushing manipulation and the elastic pad attached to the tip of the sticks in the case of the rimless wheel. In other words, the contact stiffness is a design parameter that we can pick to optimize the control performance. In the case of the pushing manipulation, we picked 100 N/m for the pusher stiffness, which produced satisfactory results, as described in the manuscript.

These are important discussion items. One paragraph in the original manuscript has been extended two paragraphs in the Discussion section.

Reviewer 2 also asked the effect of diverse contact environment conditions. The above numerical analysis of the pushing manipulation indicates that the overall MPC performance is good for the wide range of the contact stiffness, 1 N/m to 400 N/m. Therefore, the proposed CCK-MPC works for the range of the contact stiffness, if the stiffness value is known. If it interacts with an unknown situation, it is required to adapt to the environment or to be robust against the parameter changes. Such adaptation and robustness are beyond the scope of the current work, but the doctoral dissertation of an author addresses a promising method for parametric adaptation.

R2-P3. *The paper provides robust simulations and hardware experiments, but there could be more emphasis on the limitations of the method in real-world applications, especially in environments with unexpected or variable contact conditions.*

Response to *R2-P3*

This is another key question. We have extended the supplementary information so that more practical issues on real-world implementation are described, and challenges and limitations in the modeling and MPC tuning are addressed. See Supplementary Method 2.3. Furthermore, we have investigated the effect of key parameter uncertainties, focusing on the friction at the contacting surface between the slider and the floor in the manipulation task. Experimental results have been described briefly in the original manuscript, but more details are in the doctoral dissertation of an author [51], as sited in this paper. We have also extended the Koopman model to a friction-aware model where coefficient of friction is treated as an input variable to the neural network when learning observables. Combined with a parameter estimator, the friction-aware CCK-MPC can adapt to the unknown friction.

There are indeed many limitations of the method that need to be considered in real-world applications. We highlight some of those that we believe are the most relevant:

- Full state information is assumed in this work. While this is easily achieved in simulation, state estimation is far from a solved problem in practice. In our hardware example, we leveraged a motion capture setup that could record the position of the slider at approximately 120Hz. However, this highlights another limitation imposed by the practicalities of measuring the state: the dynamic behavior demonstrated by the shoving of the square slider requires high-frequency estimates of the slider's velocity. Even with the motion capture system, taking derivatives of the position to estimate the velocity can lead to noisy measurements without sufficient filtering.
- Offline data (either simulated from a model or collected from hardware) is needed to build the Koopman model. This can limit the generalizability of the approach when applied to unseen environments. In the case of unmodeled and unexpected contact environments that the Koopman model was not trained on, we would expect the method to fail, as in the case of many other data-driven methods. However, there is room for certain modeling errors to be mitigated. The feedback control provided by MPC allows for certain friction mismatches to be recovered from. See the circle slider with a friction mismatch in Fig. 6B in the original manuscript. The friction that is smaller than estimated incurs overshoots, but the MPC reactive control can manage to track the trajectory. The doctoral dissertation of an author has extended the results for the planar pusher to a method for incorporating updated friction estimates into the Koopman model, allowing for it to be generalizable to more environments without retraining.
- We would like to highlight an additional limitation: training an effective Koopman model is not trivial. A benefit of CCK-MPC is that we only have to learn an autonomous model of the dynamics, which does greatly reduce the required quantity of time and data when compared to alternatives (e.g. reinforcement learning approaches, Koopman learning for both the A and B matrices simultaneously). Nevertheless, we found empirically that poor quality data could lead to underperforming models, and that performance was sensitive to the hyperparameters of the Deep Koopman Network in the case of the planar pusher. Some of these challenges are discussed in more detail in one of the author's recent dissertations.

With these limitations in mind, we want to clarify that one of the benefits of CCK-MPC is the way that it can respond to unexpected changes in contact conditions *within the bounds of the nonlinear system* that CCK was built on. This is a major contrast to alternative linear techniques that struggle when the contact conditions change unexpectedly (say when a disturbance leads to a sudden break in contact). CCK-MPC can function even when the contact mode changes in this way. This can be seen in the simulated example of the circle slider with a lower-than-expected friction in Fig. 6B: contact is repeatedly made-and-broken unexpectedly due to the slider sliding

around the plane. Nevertheless, CCK-MPC can handle the unexpected changes in contact gracefully. In fact, CCK-MPC is structured in this work so that no specific contact mode is "expected". No bias, reference, or guidance is provided that would push the system into a desirable contact state. Rather, the CCK model is expected to completely determine the optimal contact mode on its own in real-time.

R2-P4. *The numerical results are promising, but some of the key figures, especially those related to the comparison of CCK-MPC against linear-MPC using local linearization, could benefit from clearer labels and additional context. In particular, some of the results could be more thoroughly explained, such as the specific conditions under which CCK-MPC outperforms the other methods.*

Response to *R2-P4*

Figure 4 has been revised to be clarify the experimental design. Both main text and figure caption have been revised for clarity.

Reviewer #3 (Remarks to the Author):

This paper presents the results of applying Koopman operators for contact-involved dynamics that often occur in locomotion and manipulation. The assumption of compliant contact is required. The authors claimed that the proposed CCK-MPC method is the first to establish a modeling methodology for subsuming segmented dynamics due to contact changes within a unified, globally-linear, and time-invariant system. With such a linear system, applying real-time MPC becomes available. The proposed method is validated in two specific systems: the rimless wheel locomotion system and the pushing/shoving manipulation system.

I have several comments:

R3-P1 *Applying Koopman operators for the studied systems may be new, but there has been extensive work on the systems with compliant contacts. For example, in both locomotion and manipulation, there are contact-implicit approaches that can automatically predict contact sequences [PTP-1] [PTP-2]. I just listed a few here, and the authors can find more studies from the robotics community. For compliant robot-environment interactions, there are various contact models that essentially unify segmented dynamics into one system for planning. To convince people that the method based on Koopman operators outperforms existing contact-implicit approaches, the authors need to provide more fruitful results.*

Response to *R3-P1*

The authors appreciate that the reviewer pointed out one of the key issues that must be clarified and confirmed. The key difference between our CCK modeling and those existing modeling methods in robotics literature is that CCK provides a globally linear model that is time-invariant and that allows for convex optimization. To our knowledge no prior work has achieved the globally linear modeling and convex optimization of robotic systems making and breaking contact. No local minima problem exists in our method. This globally linear modeling enabled

the real-time MPC control with an average runtime of 5.2 milliseconds for dynamic pushing and shoving tasks. This is 10~100 times faster than traditional counterparts, without the use of any bespoke hardware or software. To clarify this point, the revised manuscript emphasizes not only unified but also globally linear modeling, which results in convex optimization.

With regards to the linear complementarity approaches presented in the references provided by the reviewer [PTP-1, PTP-2], they are not applicable to real-time control; we believe that they are both intended to be motion “planners” rather than online, reactive controllers. Reference [PTP-1] is presented only with simulated results, and while computation times for the controlled ANYmal experiments are not presented, a 1s trajectory for the bouncing ball for example takes 0.5s to compute – as opposed to the <10ms goal considered in our work. Reference [PTP-2] reports solve-times of 25 minutes for the quadruped gait generation case, and as such uses iLQR to help follow the produced trajectory in practice. Nevertheless, even this iLQR controller is shown to require stabilization times in the range of 10s (see Fig. 7 [PTP-2]): 3 orders of magnitude longer than our run time, which is less than 10 ms. In this way, we argue that CCK-MPC does indeed outperform the contact-implicit approaches described in these works in the sense of online, reactive control.

In the original manuscript’s introduction, we refer to a number of existing approaches for reactive and online contact-rich tasks. Each of these approaches are powerful methods capable of performing specific tasks under specific conditions. In particular, we note the following:

a) Simplified Dynamics
    i. Quasi-dynamic or quasi-static models[1,2,8–10]
    ii. Models of reduced dimensionality and complexity[11–16]
b) Linearization at a reference point or along a reference trajectory[17]
c) Pre-defined contact mode sequences[9,10,16,18–21]
d) The use of bias terms to guide contact actions[23]

We have extended this list of related works, including the references pointed out by the reviewer [PTP-1, PTP-2]. We do not intend to claim that CCK-MPC can replace these methods wholesale, in the same way that these existing methods haven’t completely replaced one another. Each approach has its own benefits and drawbacks. However, CCK-MPC has achieved real-time, reactive control of truly dynamic contact tasks without pre-defined contact sequences, reference point or trajectory around which the process is linearized, and the use of bias terms for guiding contact actions. The globally linear Koopman modeling with the control-coherent structure has enabled convex MPC, which is an unarguable milestone.

We also cited some recent work in our manuscript that has sought to apply more advanced computational methods to speed up contact-implicit methods [23]. We are hopeful that

practitioners in our field will engineer further improvements to these methods over time, and we are excited to read about their work in future publications. It is our opinion that this does not invalidate the development of Koopman-based techniques such as CCK-KMPC, which could also stand to benefit from improved computational methods specifically geared towards them.

We also foresee future work that seeks to combine the benefits of existing methods. Consider [10], which is a recent preprint cited in our manuscript. The presented convex approach is intended for relatively long-horizon motion planning, and deliberately incorporates the seminal work in [9] as a lower-level controller. CCK-MPC could be leveraged alongside long-horizon planners in a similar way, providing rapid and reactive control to account for disturbances or modeling errors that would otherwise cause the pre-planned trajectory to fail.

R3-P2 *Going back to the proposed method, the CCK model is built using simulated data. This constrains the available systems because not all systems can be simulated easily. Assuming a system can be simulated, does the gap between the simulated system and the physical system affect the control performance? How does a user assess the availability and tolerance? Any systematic methods?*

Response to *R3-P2*

We thank the reviewer for highlighting this concern.

First, the authors would like to clarify that data-driven methods can be used for constructing a CCK model instead of relying solely on simulation. If the system is difficult to simulate, we can use experimental data. However, CCK does require basic knowledge about the system's underlying dynamics to ensure that the CCK compatibility conditions are met. See Control-Coherent Koopman Modeling under Results. That is, i) the independence of actuator and plant states must be guaranteed, and ii) the linear $\boldsymbol{B_p}$ matrix must be constructed separately prior to the identification of the $\boldsymbol{A}$ matrix either from the physical model directly or based on experiments of the actuators.

In this manuscript, we addressed how compliant contact models can guarantee the independence of actuator state for condition i), and prior work has looked at alternative scenarios, such as via the powertrain compliance of actuators [35]. That is to say, we must at least know that the system being modeled can be represented as:

$$\boldsymbol{p_{t+1}} = f_p(\boldsymbol{p_t}, \boldsymbol{q_t}) + \boldsymbol{B_p u_t} \tag{4}$$

$$\boldsymbol{q_{t+1}} = f_q(\boldsymbol{p_t}, \boldsymbol{q_t}) \tag{5}$$

In this manuscript, we assumed knowledge of f_p and f_q for the generation of training data for Koopman modeling. However, this may not be required. This is the standard Koopman modeling

problem of autonomous systems with no exogenous control input. There are many existing approaches for the data-driven construction of Koopman models, which can be readily integrated with the presented CCK approach. These approaches include DMD, eDMD and DKN techniques [27, 29, 33, 40, 47, 52, 53] as well as data-driven direct encoding [56]. Data-driven methods have also been demonstrated experimentally [48]. By leveraging these existing methods, CCK-MPC can be extended to systems for which f_p and f_q are unknown, or otherwise unable to be simulated. We also wish to mention that direct encoding methods present an approach at the opposite end of the spectrum: generating a Koopman model directly from the known nonlinear models, without the need to use simulations to generate training data [26, 54].

As for the second condition on the $\boldsymbol{B}$ matrix, ii), we determined the linear control term $\boldsymbol{B_p u_t}$ based on the physical model of the actuator mechanism in the rimless wheel case and the integrator model based on the high-fidelity velocity feedback assumption in the case of manipulation. It depends on specific cases but, in general, the actuator dynamics can be identified without difficulties as they are determined separately from the plant dynamics.

Secondly, we appreciate the reviewer's comments regarding the impact of the sim2real gap on performance, as well as on whether there are any systematic methods for addressing these issues. It is our understanding that systematic methods for overcoming the sim2real gap (and systematically evaluating its impacts) are major areas of ongoing research. The authors would like to highlight a few recent papers on the matter [PTP-4 ~ PTP-7]. To the best of our knowledge, the evaluation of the sim2real gap remains largely an empirical effort based on benchmarks and system-specific testing. However, we found that CCK-MPC could succeed even with significant gaps. Consider that the model used in the planar pushing case relied upon a simple viscous friction assumption, ignoring effects such as the Stribeck effect, static friction, and hysteresis [PTP-8]. Nevertheless, we see good performance on hardware. Reactive controllers such as CCK-MPC are well-positioned to be robust to the sim2real gap, as long as the prediction time horizon is short enough to avoid modeling errors from growing rapidly.

To address the impact of sim2real gap, it is important to conduct parameter studies. There are a few parameters that are critically important but are difficult to model. We have extended the Discussion section and added new materials to the supplementary information, Supplementary Discussion 1.2 on the contact stiffness. In the case of pushing and shoving manipulation, the friction between the slider and the floor is critically important but is difficult to determine for simulation. Figure 6d show a case where the true friction is deliberately set to be lower than that used to generate a Koopman model. The performance degrades, but the robot can still dribble the slider and push it to a goal position. More details are described in the doctoral dissertation of an author. In the dissertation, a friction-aware Koopman model is also described. This method can make the system adaptive to an unknown floor friction with the use of a parameter estimator [51].

In summary, we agree to the reviewer that sim2real gaps are critical issues and that efforts must be taken to fill the gaps. In our current work, we have a) incorporated experimental data and system knowledge to fill the gap, b) conducted parameter studies to evaluate their uncertainty upon modeling accuracy and control performance, and c) suggested an adaptive method as a future research topic.

R3-P3 *While generating simulated data, a stabilizing control input with small feedback gains is used. In this case, people may face a chicken-and-egg problem. The goal is to design a real-time MPC control scheme based on the CCK model. However, some control is required in generating data. For simple systems, we can use intuition to inject control efforts. If the system is complex, how can you ensure your controlled data generation is sufficient for determining A matrix?*

Response to *R3-P3*

Thank you for this comment, and for allowing us to clarify. When a system is unstable or marginally stable, it is difficult to generate data in both simulation and experiment. Forming a small-gain feedback loop to prevent the system from immediately diverging or escaping from a dynamic range is a well-established practice in system identification [PTP-9, PTP-10]. It has been widely used and is effective. Such small-gain feedback may be simple proportional control, PID, or ones with feedforward compensation. In applying this technique, the proposed CCK modeling method has an important advantage because the control term of the system appears linearly. We can simply subtract the effect of the feedback control from the simulation and/or experimental data. As the reviewer pointed out, this data manipulation is difficult if the control is nonlinearly involved. However, the CCK modeling approach facilitates it, being another feature of the proposed method.

R3-P4 *I understand the goal of this paper may be to share a paradigm, so the studied systems are specific and relatively simple/intuitive, and the resulting performance is predictable and not beyond the existing. However, for future users like researchers in the robotics community, we need more generic and deployable approaches. For example, contact-implicit trajectory optimization may suffer from some limitations, but general enough for all contact-involved systems. Researchers can follow it for their own study with improvement and make systems work, which is highly meaningful for robotics. At this moment, the proposed method is only validated on several simple systems, and no further confidence can be provided to other researchers, considering A matrix is determined by simulated data, and most on-going studies involve complex systems like legged manipulators with both locomotion and manipulation in the same system.*

Response to *R3-P4*

The authors disagree to the reviewer's statement, "the proposed CCK-MPC method is not general enough". The CCK formulation is theoretically rigorous and is built upon a solid mathematical foundation. Modeling properties 1) and 2) and conditions a) and b) clearly and rigorously stipulate under which conditions the method is applicable. See the Control-Coherent Koopman Modeling subsection in the Results section. These are all general conditions that

enable engineers to examine whether the method is applicable to their problems. The method was validated theoretically and not by examples.

Furthermore, the reviewer's comment, ".. and the resultant performance is… not beyond the existing" does not reflect the truth. The paper has accomplished the tasks that are far beyond the existing works. In particular, the CCK-MPC of pushing and shoving manipulation has gone clearly beyond the current limit. No prior work could achieve truly dynamic 2D pushing and shoving (the traditional counterpart is quasi-static and quasi-dynamic); we have solved the trajectory optimization problem without a pre-determined sequence of contact changes or a reference trajectory; and we have achieved the real-time MPC control with an average runtime of 5.2 ms (over 10 times faster). This is a clear breakthrough. Furthermore, the manuscript rigorously addressed how and why these unprecedented accomplishments were made possible.

Our intention with this manuscript is to present the theoretical foundation and initial demonstrated results to develop a Koopman-based method for working with controlled, contact-based systems. It is our hope that based on our foundational research other researchers will be able to apply and improve upon. For example, we would be interested to see how groups studying the use of Koopman models for controlling soft robots [48] may use CCK-MPC to introduce contact into their systems. We also believe that various methods for generating Koopman models of autonomous systems can be combined with our CCK-MPC formulation for more rigorously applying the Koopman theory to non-autonomous systems and, thereby, improving prediction accuracy and control performance. We would be excited to see how stable Koopman approaches may be applied to the proposed method [PTP-11] (we specifically want to highlight the work by Mamakoukas et al. on DISKO, which they demonstrated on a slower-than-real time planar pushing example with a predefined contact trajectory – an indication that their work may be an especially exciting avenue of further study for CCK-MPC). Other methods of producing the Koopman observables, such as Hankel-based observables [PTP-12], could also be applied to CCK-MPC. The theoretical foundation of the CCK formulation given in this manuscript will guide those authors to address how CCK can be applied to and combined with their methods, and the exemplary case studies in robot locomotion and dynamic manipulation will inspire them.

R3-P5: *Going back to the validation provided in this paper, there is no physical validation for the rimless wheel system. Physical experiments are crucial for robotics because implementation problems will arise while deploying your algorithms onto a robot. If the authors do not make this effort, it would be even harder for other researchers who are new to the authors' methodology. Overall, the exploration in this paper is interesting. However, there are more efforts to be made before it is useful and impactful to the robotics community.*

[1] I. Chatzinikolaidis, Y. You and Z. Li, "Contact-Implicit Trajectory Optimization Using an Analytically Solvable Contact Model for Locomotion on Variable Ground," in IEEE Robotics and Automation Letters, vol. 5, no. 4, pp. 6357-6364, Oct. 2020.

[2] V. Kurtz and H. Lin, "Contact-Implicit Trajectory Optimization with Hydroelastic Contact

and iLQR," 2022 IEEE/RSJ International Conference on Intelligent Robots and Systems (IROS), Kyoto, Japan, 2022, pp. 8829-8834.

Response to *R3-P5*

The authors agree that physical validation is important. That is why the dynamic pushing and shoving manipulation has been fully implemented on a physical robot and real experimental results have been presented. Furthermore, various practical issues in implementing the proposed CCK-MPC method have been addressed. Repeating the same for the active rimless wheel is a) unnecessary and b) infeasible due to the word limit.

We would like to point out that the two papers suggested by the reviewer [PTP-1, PTP-2] have limited hardware experiments themselves; those examples are primarily simulation studies. If the suggested papers represent a common practice, including a case study / example having no physical validation is acceptable as long as one robot system is fully implemented and experimental results are presented.

Having said that, the authors still think it important to implement theoretical results on a physical robot. The authors' laboratory has been developing an actuated rimless wheel, as shown in the photo below. Building the right prototype and conducting experiments are challenging tasks. In engineering, details are important. Presenting these engineering details is beyond the scope of the current work, and there is no enough space to include the engineering details, including design and control implementation. Also, it is not in the interest of the readership of a science journal like Nature Communications. We plan to publish the design, fabrication, control implementation, and test of the rimless wheel in an engineering conference / journal.

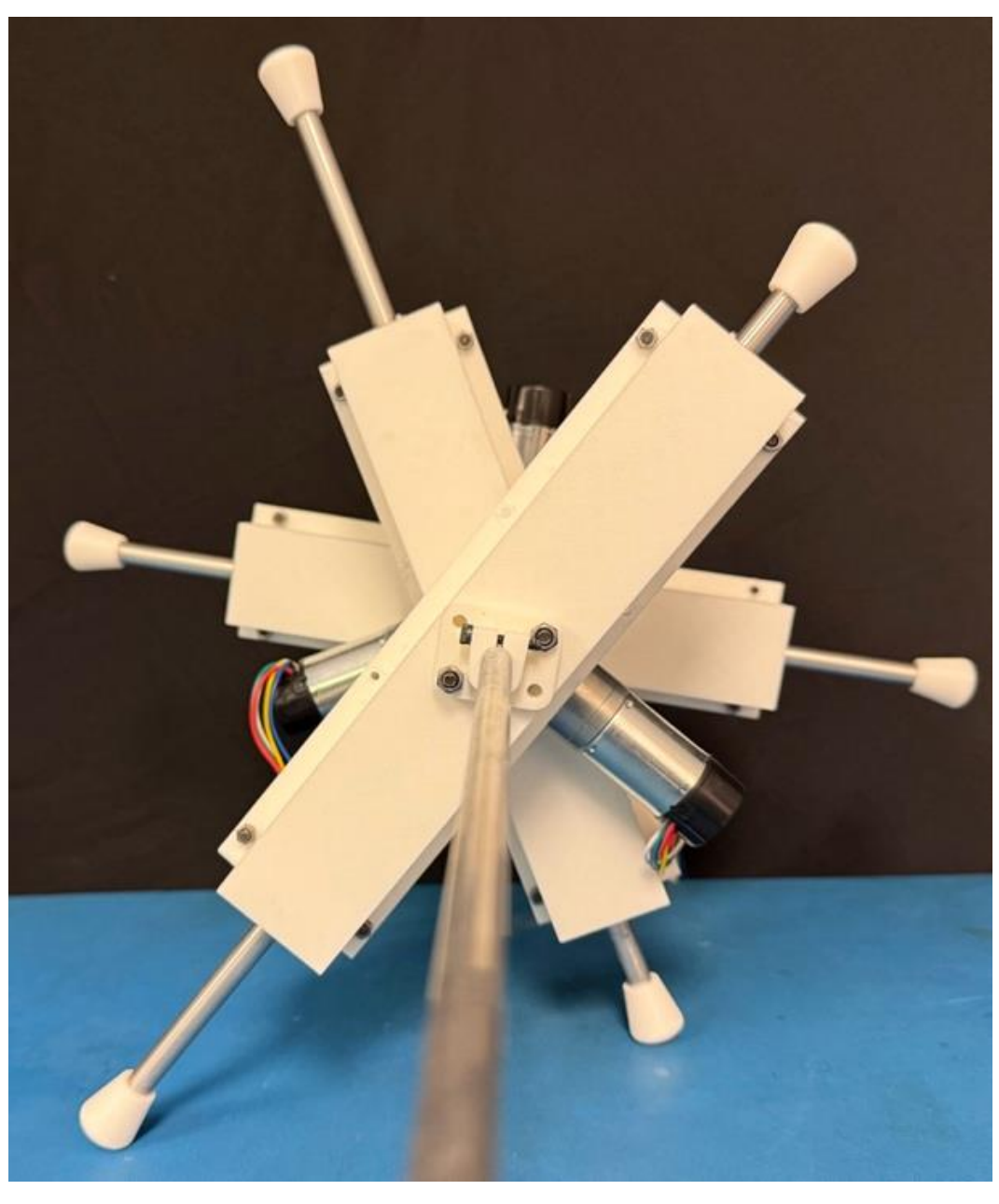

PTP Fig.7 Actuated rimless wheel prototype

References

[PTP-A] Asada, H.H., “An Actuator Pre-Filtering Approach to Control-Coherent Koopman Modeling: Extending Koopman Operators to Systems with Control”, IEEE CONTROL SYSTEMS LETTERS, VOL. 9, 2025.

[PTP-1] I. Chatzinikolaidis, Y. You and Z. Li, "Contact-Implicit Trajectory Optimization Using an Analytically Solvable Contact Model for Locomotion on Variable Ground," in IEEE Robotics and Automation Letters, vol. 5, no. 4, pp. 6357-6364, Oct. 2020. – Suggested by Reviewer 3

[PTP-2] V. Kurtz and H. Lin, "Contact-Implicit Trajectory Optimization with Hydroelastic Contact and iLQR," 2022 IEEE/RSJ International Conference on Intelligent Robots and Systems (IROS), Kyoto, Japan, 2022, pp. 8829-8834. – Suggested by Reviewer 3

[PTP-3] Q. Le Lidec, W. Jallet, L. Montaut, I. Laptev, C. Schmid and J. Carpentier, "Contact Models in Robotics: A Comparative Analysis," in IEEE Transactions on Robotics, vol. 40, pp. 3716-3733, 2024, doi: 10.1109/TRO.2024.3434208.

[PTP-4] Q. Le Lidec, W. Jallet, L. Montaut, I. Laptev, C. Schmid and J. Carpentier, "Contact Models in Robotics: A Comparative Analysis," in IEEE Transactions on Robotics, vol. 40, pp. 3716-3733, 2024, doi: 10.1109/TRO.2024.3434208.

[PTP-5] D. Blanco-Mulero, O. Barbany, G. Alcan, A. Colomé, C. Torras and V. Kyrki, "Benchmarking the Sim-to-Real Gap in Cloth Manipulation," in IEEE Robotics and Automation Letters, vol. 9, no. 3, pp. 2981-2988, March 2024, doi: 10.1109/LRA.2024.3360814.

[PTP-6] Sudhakar, Sruthi, et al. "Exploring the sim2real gap using digital twins." *Proceedings of the IEEE/CVF International Conference on Computer Vision*. 2023.

[PTP-7] A. Kadian *et al*., "Sim2Real Predictivity: Does Evaluation in Simulation Predict Real-World Performance?," in *IEEE Robotics and Automation Letters*, vol. 5, no. 4, pp. 6670-6677, Oct. 2020, doi: 10.1109/LRA.2020.3013848.

[PTP-8] Anitescu, M., Potra, F.A. Formulating Dynamic Multi-Rigid-Body Contact Problems with Friction as Solvable Linear Complementarity Problems. *Nonlinear Dynamics* **14**, 231–247 (1997). https://doi.org/10.1023/A:1008292328909

[PTP-9] L. Ljung, “System Identification: Theory for the User, Second Edition”, Prentice-Hall, (1999).

[PTP-10] Jer-Nan Juang, “Applied System Identification”, Prentice Hall, (1994).

[PTP-11] G. Mamakoukas, I. Abraham and T. D. Murphey, "Learning Stable Models for Prediction and Control," in *IEEE Transactions on Robotics*, vol. 39, no. 3, pp. 2255-2275, June 2023, doi: 10.1109/TRO.2022.3228130

[PTP-12] Brunton, S.L., Brunton, B.W., Proctor, J.L. *et al.* Chaos as an intermittently forced linear system. *Nat Commun* **8**, 19 (2017). https://doi.org/10.1038/s41467-017-00030-8

Point-by-Point Response to the Second-Round Reviewers' Comments

NCOMMS-25-48772

Koopman Global Linearization of Contact Dynamics for Robot Locomotion and Manipulation Enables Elaborate Control

All the reviewers' comments are reproduced verbatim and are shown below in *Italic*, while the authors' response is written in a regular font.

Each point raised by the reviewers is labeled as ***Rx-Py***, meaning that Reviewer *x*, Point *y*.

Reviewer #1 (Remarks to the Author):

R1-P1. *The authors provided a substantive rebuttal with ample examples that directly respond to my comments. I look forward to the published version of the paper with the provided example.*

Response to *R1-P1*

We would like to thank the reviewer for raising critical issues and giving us the opportunity to address them properly with additional results. We are pleased that the reviewer appreciated our revised paper and response. We hope that the published work lives up to their expectations.

Reviewer #2 (Remarks to the Author):

R2-P1. *The paper has been improved significantly. I just have one more suggestion.*

The comparison with bilinear MPC is great. It would also be very convincing to compare your method's performance and speed against a more standard approach, like an MPC that uses local linearization along a pre-planned trajectory (LTV-MPC).

Response to *R2-P1*

Thank you for the suggestion. We agree that additional comparisons with local linearization methods would strengthen the manuscript. We recorded the performance of KMPC, Time Varying Linear MPC (TVLMPC), and Linear MPC (LMPC) when tasked with pushing the circular slider towards a fixed goal. This comparison was performed across a set of 45 initial conditions for each of the linearization approaches. This additional result has been added to the Supplementary information.

In our implementation, we made the following distinction between TVLMPC and LMPC:

LMPC – a linearization is created from the initial state of the pusher-slider system and used throughout the task.

TVLMPC – at each time step, the current state of the pusher-slider system is used to perform a local linearization. This ensures that the local linear model is as accurate as possible within each

discretized time step. However, the system accumulates errors due to its inability to plan across mode boundaries, since the linearized A matrix is kept constant during each iteration of MPC.

Our results indicate that KMPC can outperform both local linearization methods in terms of control performance. This is especially the case with regards to the tail-end of the distribution of our test set, where contact state must be changed. See Figure 1A below. KMPC's sum of squared error is an order-of-magnitude smaller than its counterparts.

In terms of computation time, we unsurprisingly find that LMPC is the fastest of the linear methods. While TVLMPC is the slowest due to the need to recompute the linear matrix in each time step, it is possible that the use of a dedicated automatic differentiation library could improve its computational speed. It is also possible that there is a more efficient way to structure the convex optimization problem with varying A and B matrices than what was implemented in this test. Given this, we caution against drawing too strong of a conclusion with regards to the comparative speed of KMPC and TVLMPC from our results.

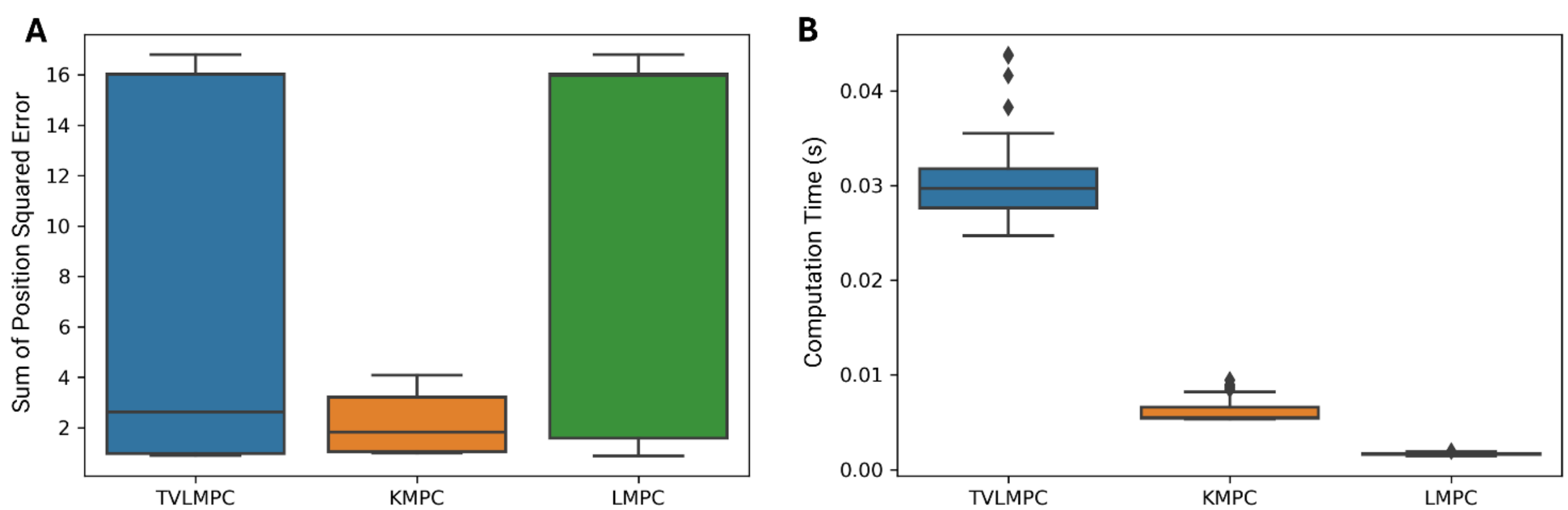

Figure 1 Comparison between KMPC and Local Linearization Methods. *The KMPC method presented in this work is compared against two alternative linear methods: Time Varying Linear MPC (TVLMPC) and Linear MPC (LMPC). (**A**) KMPC outperforms TVLMPC and LMPC in terms of control performance for the given task. This is especially the case in the tail end of the test distribution, with both TVLMPC and LMPC underperforming with more challenging initial conditions. (**B**) A key benefit of LMPC with a constant linear model is the fast computation enabled during convex optimization. Nevertheless, KMPC is still able to achieve sufficiently fast times of under 10ms.*

Reviewer #3 (Remarks to the Author):

The reviewer raised their concerns about the proposed Koopman modeling capability, practicality, scalability, and sim-to-real gaps. Many of these concerns apply to broad machine learning and data-driven approaches in general. Fully addressing these is beyond the scope of a single paper. However, the authors agree with the reviewer that those challenges are present and that we must more clearly state these limitations. We have added the following paragraph to the

end of the Discussion section for manifesting the reviewer's concerns and emphasizing the needs for future work.

> "Beyond the effect of ablation, applying the current Koopman modeling to practical problems remains a challenge when the method is applied to high-order systems having significant sim-to-real gaps and difficulties in collecting effective data, among others. These are not necessarily limitations specific to the proposed CCK-MPC framework but are challenges common to machine learning and data-driven methods. While ongoing research efforts in Koopman and machine learning communities will remove some limitations, significant work must be done to address all these issues. These efforts will enhance CCK-MPC and, in turn, CCK-MPC complements ongoing efforts by providing a methodology for creating linear model that subsumes segmented contact models and enables convex optimization."

The following Point-by-Point responses address specific issues, clarify the reviewer's concerns, and make the arguments accurate and rational.

R3-P1-a. *My original concern was not about runtime but about modeling capability and relevance. The authors' reply focuses mainly on computational speed, which does not address whether the proposed Koopman-based approach actually improves the modeling of compliant or contact-rich dynamics compared with the extensive literature on contact-implicit and compliant-contact methods.*

Response to *R3-P1*-a

Runtime is a critical metric for evaluating the effectiveness of algorithms and methods for practical, real-world applications. However, this paper addresses more fundamental features and advantages attained with the proposed CCK-MPC. Among others, "convexity" of optimization is the most significant feature that fundamentally changes the computational framework. It guarantees the optimality of a solution obtained from a straightforward computation, while other methods cannot guarantee the optimality or must employ more complex techniques. We use runtime as a critical metric, but the core contribution comes from profound theoretical issues.

We would also like to clarify that it is not our intention to detract from the capabilities of the existing methods, such as contact-implicit and compliant-contact methods. Similarly, this paper is not on improving the contact model, rather in using existing contact models to generate Koopman representations of the dynamics that are useful for real-time control. The contact model used to generate the Koopman model can be those described in the paper or from high fidelity physics simulators like MuJoCo or Drake, or from more extensive experiments. Our focus is to use these contact models and convert them to a dynamic representation amenable for convex MPC and real-time control.

R3-P1-b. *Moreover, the presented results are limited to simple, low-dimensional systems, which makes the claimed speed advantage less compelling to a broader robotics audience concerned with scalability to manipulation, locomotion, or whole-body control. Without evidence that CCK maintains accuracy or generality on more realistic multi-contact tasks, or comparative evaluations against established contact-implicit frameworks, it remains difficult to assess whether the proposed method offers meaningful advantages beyond faster inference on toy examples.*

Response to *R3-P1-b*

We would like to clarify some of the above statement and make it more accurate. With regard to the phrase "a toy problem", we do not think that the non-prehensile pushing task is merely a toy problem; it has been a popular benchmark in the contact-rich manipulation literature [PTP-1], [PTP-2], [PTP-3]. This benchmark problem has never been solved satisfactorily without making restrictive assumptions, as addressed in the Introduction section. Based on the CCK-MPC formulation, we have substantially extended it to dynamic pushing and shoving. The rimless wheel, too, is not a simple problem. The order of the system (the number of independent state variables) is 18; with symmetricity, we have reduced the order to 12. Nonetheless, the problem is not a low-order system. Along the same line, we must disagree with the reviewer's statement that initial implementation with simpler systems is irrelevant. This is especially the case when developing new methodologies. The exemplary case studies we addressed in this paper are at the right level of complexity, which are not trivially low-order but are transparent and accountable for verifying the new methodology.

On the other hand, we agree with the reviewer's point that demonstrating CCK-MPC on more complex, multi-contact tasks is a worthwhile goal. We have added a paragraph to the discussion section, stating that "applying the current Koopman modeling to practical problems remains a challenge when the method is applied to high-order systems having significant sim-to-real gaps and difficulties in collecting effective data,…." It is desired to extend it to a higher order, as the reviewer stressed. There is a large area of research focused on making Koopman models more scalable by leveraging machine learning techniques. For example, a very recent paper already demonstrated Koopman models for high-dimensional, contact-rich systems. The paper published during the review process of our paper applied Koopman operators to legged robots with higher order [PTP-4]. We must emphasize, however, that although extending the Koopman modeling to higher-order systems is important, it is not a key focus of our paper. Our paper emphasizes the control-coherent Koopman modeling, which has been missing in the Koopman modeling literature. The recent paper mentioned above [PTP-4], too, uses DMDc to generate its B matrix and the MPC computation is not completed in real time. Control-coherent B matrix formulation is critically important in applying the Koopman-based modeling to robots and all systems with control input.

Another point to clarify is complexity of segmented systems. As the number of contact modes and the number of contact points increase, the model complexity increases. Optimization of contact sequence, for example, increases exponentially as the number of contact modes and contact points increases. However, the complexity of the resultant Koopman representation does not directly depend on the number of contact modes, i.e, the number of segmented regions. As the number of contact points and contact modes increases, the number of segmented regions increases, but those segmented dynamics are convoluted and integrated into a Koopman operator. The result is a linear matrix amenable for real-time control. This implies that the Koopman approach has the potential to deal with contact-rich systems.

R3-P2-a. *While I appreciate the authors' expanded discussion, the response still does not fully address my underlying concern. My question was not simply about whether data-driven methods can be used, but about the practical availability and reliability of constructing CCK models for real systems that cannot be easily simulated or modeled.*

Response to *R3-P2-a*

Thank you for the feedback. To address the reviewer's first point, Koopman models and the CCK method can be used for systems that cannot be easily simulated or modeled. In many applications, a Koopman model is obtained through data alone, without requiring explicit equations of motion. Some of the most influential work in the Koopman literature originated in the fluid dynamics community, where Koopman models were learned directly from experimental flow and velocity data [PTP-5]. Koopman modelling is arguably best suited for systems such as soft robots, where analytical models are unknown [PTP-6], or systems governed by PDEs with highly complex dynamics. In summary, it is known that the Koopman modeling method is applicable to "real systems that cannot be easily simulated or modeled".

R3-P2-b. *The reply lists candidate data-driven Koopman techniques and acknowledges that sim-to-real gaps remain largely empirical, but it does not provide clear guidance on how a user should assess when a system satisfies the CCK compatibility conditions, how much sim-to-real discrepancy can be tolerated before control performance degrades, or whether any systematic diagnostic methods exist for evaluating model adequacy. Therefore, while the response provides useful context, it does not fully answer the original question regarding the practical feasibility and robustness of constructing CCK models for systems that are not straightforward to simulate or whose dynamics contain significant uncertainty.*

Response to *R3-P2-b*

The key compatibility condition / assumption is the contact compliance. We have addressed this quantitatively. As presented in the Supplementary Discussion 1.2 and Figure 6, we have analyzed the effect of the stiffness at contact points upon modeling accuracy and control performance. To address the sim-to-real gap, we have examined the floor friction in the pushing

and shoving tasks. We have also cited an author's doctoral thesis describing details about the sim-to-real gap of floor friction.

We did not propose general tools and methodologies, which are beyond the scope of the current work. To the best of our knowledge, there are scarce system diagnostic methods for evaluating this problem across data-driven modeling techniques, and we do not introduce one for CCK modeling within this work [PTP-7]. However, these limitations are not unique to the proposed CCK modelling method, but are shared broadly across the Koopman and machine learning communities [PTP-8]. One practical approach to mitigating the sim-to-real gap is augmenting simulation-trained models with data collected from hardware. Additionally, useful diagnostic measures exist to determine Koopman model validity. One such measure is Koopman invariance, which evaluates whether lifted states are mapped consistently using the learned operator [PTP-9]. Applying these methods to the CCK formulation and delving deeper into the subject will create a new paper but are not within the scope of the current work as they are not its main contribution.

Finally, robustness to simulator/parameter mismatch is an active area of research in our lab. Preliminary results indicate that explicitly incorporating system parameters into the Koopman lifting can yield models that generalize for different parameters such as friction coefficients, mass, and inertia. In such cases, online parameter estimation can be used during runtime to adapt the model to varying deployment conditions.

R3-P3. *Thank you for the detailed clarification, but my concern is not about the theoretical rigor of the CCK formulation, but about its practical generality and deployability for future users in the robotics community. The mathematical conditions for CCK applicability are helpful, but they do not substitute for evidence that the approach can scale to the kinds of high-dimensional, hybrid, contact-rich systems that are common in robotics research. At present, the method has only been demonstrated on relatively simple and low-dimensional examples; thus, it remains difficult for users to judge whether CCK-MPC will behave reliably, remain tractable, or maintain modeling fidelity on more complex platforms. Contact-implicit trajectory optimization, despite its limitations, can be used because it is general enough for arbitrary contact-involved systems and provides a deployable pipeline for diverse tasks. Without broader validation or benchmarks on more challenging real-world systems, it is hard to conclude that CCK-MPC offers general advantages or can serve as a practical tool beyond the specific examples shown.*

Response to *R3-P3*

The practical applicability, including the key conditions for the CCK formulation and the sim-to-real gap in practical implementation, have been addressed in the analysis of the effect of contact compliance and the floor friction. Both are specific to the CCK formulation and presented for the provided case studies. We have completed the whole process of physical modeling, data collection, simulation, model validation, and control design for the case studies. Beyond the specific CCK-MPC issues, fully addressing general issues in broad Koopman

modeling and machine learning fields is not the objective of the current work, but the importance of practical implementations has been acknowledged in the Discussion section, and a number of relevant references have been cited.

The exemplary case studies dealt with systems with up to 18 (12 after leveraging symmetry) independent state variables. These are not trivially simple, low-order systems, but are at the right level of complexity for presenting a new methodology. As stated previously, a recent paper that came out during this review process dealt with a high-dimensional locomotion system, indicating the possibility of applying the Koopman to more complex systems.

Contact-implicit MPC has many crucial advantages – as the reviewer alluded to in their earlier feedback. This is achieved by leveraging linear complementarity constraints in explicit models of the system-of-interest's dynamics. For cases where these constraints cannot be readily applied, data-driven techniques may be more effective [PTP-2]. As the reviewer acknowledged, contact-implicit MPC has known limitations and drawbacks. It is not our intention to present CCK-MPC as a cure-all for the challenge of contact-rich manipulation, but we do believe that the method will be a useful addition to the robotics practitioner's toolkit, alongside alternative approaches.

R3-P4. *In summary, while the CCK formulation is theoretically elegant, the authors' responses do not fully resolve concerns about its practical relevance, scalability, and usability for real-world robotic systems. The current validation is confined to simple, low-dimensional examples, and the replies emphasize computational speed and theoretical generality without providing concrete evidence that CCK can robustly model or control the complex and contact-rich dynamics encountered in modern robotics. Moreover, questions regarding how users should assess applicability, manage sim-to-real discrepancies, and reliably construct CCK models for systems that are difficult to simulate remain insufficiently addressed. As a result, it is still unclear whether the proposed approach can serve as a broadly deployable tool for the robotics community.*

Response to *R3-P4.*

These concerns have been addressed in the previous responses. Specifically, the reviewer raises three issues: (1) applicability to high-DOF, contact-rich systems; (2) sim-to-real discrepancies and the difficulty of quantifying model error; and (3) the feasibility of constructing CCK models for systems that are difficult to simulate or model analytically. These are very important concerns with regards to the overall usefulness of our proposed method, and while we are confident that CCK models can be constructed for many systems with unknown dynamics, the first two points are open questions within the broader Koopman and learning-based robotics communities. Addressing them comprehensively is beyond the scope of a single paper.

In this work, we introduce a method for correctly incorporating a time-invariant control input matrix into Koopman models, enabling linear time-invariant representations of nonlinear

systems with contact for use in real-time control. Convexity in MPC optimization has had a significant impact upon otherwise extremely challenging real-time MPC. Our proposed approach inherits limitations common to Koopman-based and machine learning methods, and there is a significant amount of work to be done to address these shortcomings. However, our method directly addresses a key bottleneck in real-time MPC and in the use of Koopman operator theory to systems with control. CCK provides a necessary modeling component that complements ongoing efforts in extending Koopman methods to complex, contact-rich robotic systems.

References

[PTP-1] F. R. Hogan and A. Rodriguez, "Feedback Control of the Pusher-Slider System: A Story of Hybrid and Underactuated Contact Dynamics," in *Algorithmic Foundations of Robotics XII*, vol. 13, K. Goldberg, P. Abbeel, K. Bekris, and L. Miller, Eds., in Springer Proceedings in Advanced Robotics, vol. 13. , Cham: Springer International Publishing, 2020, pp. 800–815. doi: 10.1007/978-3-030-43089-4_51.

[PTP-2] N. Dengler, J. Del Aguila Ferrandis, J. Pousa De Moura, S. Vijayakumar and M. Bennewitz 2025, "Learning Goal-Directed Object Pushing in Cluttered Scenes With Location-Based Attention" in *Proceedings of the 2025 IEEE/RSJ International Conference on Intelligent Robots and Systems*. Proceedings of the International Conference on Intelligent Robots and Systems, pp. 1-7.

[PTP-3] T. Pang, H. J. T. Suh, L. Yang, and R. Tedrake, "Global Planning for Contact-Rich Manipulation via Local Smoothing of Quasi-Dynamic Contact Models," *IEEE Transactions on Robotics*, vol. 39, no. 6, pp. 4691–4711, Dec. 2023, doi: 10.1109/TRO.2023.3300230.

[PTP-4] F. Li, A. Abuduweili, Y. Sun, R. Chen, W. Zhao, and C. Liu, "Continual Learning and Lifting of Koopman Dynamics for Linear Control of Legged Robots," in *Proceedings of the 7th Annual Learning for Dynamics \& Control Conference*, PMLR, May 2025, pp. 136–148. Accessed: Jan. 08, 2026. [Online]. Available: https://proceedings.mlr.press/v283/li25b.html

[PTP-5] P. J. Schmid, "Dynamic mode decomposition of numerical and experimental data," *Journal of Fluid Mechanics*, vol. 656, pp. 5–28, Aug. 2010, doi: 10.1017/S0022112010001217.

[PTP-6] "A Koopman-based residual modeling approach for the control of a soft robot arm - Daniel Bruder, David Bombara, Robert J Wood, 2025." Accessed: Jan. 07, 2026. [Online]. Available: https://journals.sagepub.com/doi/10.1177/02783649241272114

[PTP-7] R. Strässer, K. Worthmann, I. Mezić, J. Berberich, M. Schaller, and F. Allgöwer, "An overview of Koopman-based control: From error bounds to closed-loop guarantees," *Annual Reviews in Control*, vol. 61, p. 101035, 2026, doi: 10.1016/j.arcontrol.2025.101035.

[PTP-8] E. Aljalbout *et al.*, "The Reality Gap in Robotics: Challenges, Solutions, and Best Practices," Dec. 2025, doi: 10.1146/annurev-control-031924-100130.

[PTP-9] J. S. Kim, Y. S. Quan, and C. C. Chung, "Uncertainty Quantification of Autoencoder-Based Koopman Operator," in *2024 American Control Conference (ACC)*, Jul. 2024, pp. 4631–4636. doi: 10.23919/ACC60939.2024.10644800.
