# Supplementary material for: Koopman global linearization of contact dynamics for robot locomotion and manipulation enables elaborate control
Source: Nat Commun. 2026 Jun 19;17:7749. doi: 10.1038/s41467-026-72485-7 (PMC13434789; doi:10.1038/s41467-026-72485-7)
Supplement: Supplementary file 2 [file 41467_2026_72485_MOESM1_ESM.pdf]

Koopman global linearization of contact dynamics for robot locomotion and manipulation enables elaborate control

C. O'Neill, J. Terrones, H. H. Asada

Contents

Supplementary Discussion 2
1.1 A Brief Introduction to Koopman Operators 2
1.2: Effect of Contact Stiffness on MPC Control Performance for Dynamic Pushing 3
1.3: Koopman Modeling of a Cart-Pole with Walls: A Canonical Example 4
1.4: Additional Comparisons of CCK-MPC to Bilinear Koopman MPC and L-MPC Approaches 6
1.5: Effect of Ablation of Koopman Observables 9
Supplementary Methods 10
2.1: Computation of **A** and **B** Matrices for the Rimless Wheel 10
2.2: Rimless Wheel MPC Formulation 11
2.3: Additional Information for Implementing CCK-MPC on the Planar Pusher 13
2.3.1 Square Slider 13
2.3.2 Circle Slider 13
Supplementary Figures 14
Supplementary Tables 15
Supplementary References 17

Supplementary Figures

Supplementary Figure 1. Impact of Varying Pusher Stiffness on Square Slider Control Performance 4
Supplementary Figure 2. Comparison of Koopman LQR vs LQR for Cart-Pole with Walls 6
Supplementary Figure 3. Results from Comparing CCK-MPC with a Bilinear Koopman MPC Method 7
Supplementary Figure 4. Comparison between KMPC and Local Linearization Methods for the Circular Slider 8
Supplementary Figure 5. Impact of Model Size on Koopman MPC 9
Supplementary Figure 6. Pseudocode Algorithm for the Planar Pusher. 15

Supplementary Tables

Supplementary Table 1. Parameter values for the Rimless Wheel Dynamics 15
Supplementary Table 2. Parameter values for the Rimless Wheel Koopman models and Simulation 16
Supplementary Table 3. DKN Hyperparameters 16
Supplementary Table 4. CCK-MPC Parameters for Planar Pushing Q 16
Supplementary Table 5. Comparison of Linearization Methods for use in L-MPC 16

Supplementary Discussion

Supplementary Discussion 1.1 A Brief Introduction to Koopman Operators

Consider a discrete-time, autonomous nonlinear system with no exogenous input:

$$\boldsymbol{x}_{t+1} = F(\boldsymbol{x}_t) \tag{1}$$

where $\boldsymbol{x}_t \in X \subset \mathbb{R}^n$, and function F is a self-map, $F: X \mapsto X$, which is continuous.

Let $\{\varphi_i(\boldsymbol{x}_t)\}_{i=1}^{\infty}$ be orthonormal basis functions that span a Hilbert space $\mathcal{H}$. Let $\varphi_i \circ F$ be the composition of the basis function φ_i with the self-map F, that is, $(\varphi_i \circ F)(\boldsymbol{x}_t) = \varphi_i(F(\boldsymbol{x}_t))$. If each function $\varphi_i \circ F$ is involved in the Hilbert space,

$$\varphi_i \circ F \in \mathcal{H}, \quad i = 1,2,\cdots \tag{2}$$

then the function $\varphi_i \circ F$ can be expanded to a series of the orthonormal basis functions $\{\varphi_i(\boldsymbol{x}_t)\}_{i=1}^{\infty}$.

$$\varphi_i \circ F = \sum_{j=1}^{\infty} \langle \varphi_i \circ F, \varphi_j \rangle \varphi_j, \qquad i = 1,2,\cdots \tag{3}$$

where $\langle -, - \rangle$ represents the inner product of two functions.

Example**:** Consider trigonometric functions, which are orthonormal basis functions. The above equation is a familiar Fourier series expansion of function $\varphi_i \circ F$.

Note that, from Supplementary Equation (1), we find that $\varphi_i(F(\boldsymbol{x}_t)) = \varphi_i(\boldsymbol{x}_{t+1})$. Namely, the function $\varphi_i \circ F$ evaluated at state $\boldsymbol{x}_t$ represents the orthonormal functions evaluated at the next state $\boldsymbol{x}_{t+1}$. Now consider the state $\boldsymbol{x}_t$ viewed from the infinite number of basis functions, called observables, $\breve{\boldsymbol{z}}_t = (\varphi_1(\boldsymbol{x}_t), \varphi_2(\boldsymbol{x}_t), \cdots)^T$. From Supplementary Equation (3), we can find that

$$\breve{\boldsymbol{z}}_{t+1} = \breve{\boldsymbol{A}}\breve{\boldsymbol{z}}_t \tag{4}$$

where

$$\breve{\boldsymbol{A}} = \begin{bmatrix} \langle \varphi_1 \circ F, \varphi_1 \rangle & \langle \varphi_1 \circ F, \varphi_2 \rangle & \cdots \\ \langle \varphi_2 \circ F, \varphi_1 \rangle & \langle \varphi_2 \circ F, \varphi_2 \rangle & \cdots \\ \vdots & \vdots & \ddots \end{bmatrix}. \tag{5}$$

Representing the state with the infinite number of observables, $\breve{\boldsymbol{z}}_t$, we can interpret Supplementary Equation (4) as a state transition equation in the infinite dimensional space. Note that this state transition is "globally" linear because the matrix $\breve{\boldsymbol{A}}$ is constant. This is strikingly different from the

standard pointwise linearization, which is valid only in the vicinity of a reference point.

While the orthonormal basis functions were used for representing the state, it can be shown that more general basis functions can be used for the state transition, if the basis functions span the Hilbert space.

$$\boldsymbol{z}_{t+1} = \mathbf{A}\boldsymbol{z}_{\boldsymbol{t}} \tag{6}$$

where $\boldsymbol{z}_t = (g_1(\boldsymbol{x}_t), g_2(\boldsymbol{x}_t), \cdots)^T$ is the infinite-dimensional state lifted with observables $\{g_i\}_{i=1}^{\infty}$, which are not necessarily orthonormal but are independent[1].

Post-multiply $\boldsymbol{z}_t{}^T$ to both sides of Supplementary Equation (6) and integrate them over the dynamic range of the independent state yields

$$\mathbf{Q} = \mathbf{AR} \tag{7}$$

where

$$\mathbf{Q} = \langle \boldsymbol{z}_{t+1}, \boldsymbol{z}_t^T \rangle = \left\{ \int_X g_i[F(\boldsymbol{x})] \cdot g_j(\boldsymbol{x}) d\boldsymbol{x} \right\} \tag{8}$$

$$\mathbf{R} = \langle \boldsymbol{z}_t, \boldsymbol{z}_t^T \rangle = \left\{ \int_X g_i(\boldsymbol{x}) \cdot g_j(\boldsymbol{x}) d\boldsymbol{x} \right\} \tag{9}$$

The Koopman operator is the solution to the linear equation Supplementary Equation (7). This method for obtaining the Koopman operator is called Koopman Direct Encoding[1]. Finding an effective set of observables is a challenge, but deep learning has been used for constructing them from data[2].

Supplementary Discussion 1.2: Effect of Contact Stiffness on MPC Control Performance for Dynamic Pushing

CCK-MPC for contact-rich systems leverages the compliance between contacting bodies to enable Koopman linearization methods. This supplementary section briefly presents initial results on how varying the contact stiffness of a system can impact the performance of a CCK-MPC controller. These results were collected by considering a numerical simulation of the planar pushing system with a square slider. A different Koopman model was trained for each stiffness value using the DKN parameters presented in Supplementary Table 3. Using the parameters from Supplementary Table 4, CCK-MPC controllers for each stiffness value were then evaluated on a task tracking a fixed-velocity straight-line trajectory in the positive x direction. The controllers were each tested with five different initial conditions, and the resultant trajectory cost (in terms of position squared error) was normalized by subtracting it from the cost of a policy that took no action. This normalization was done to verify that the CCK-MPC controllers met some basic level of performance.

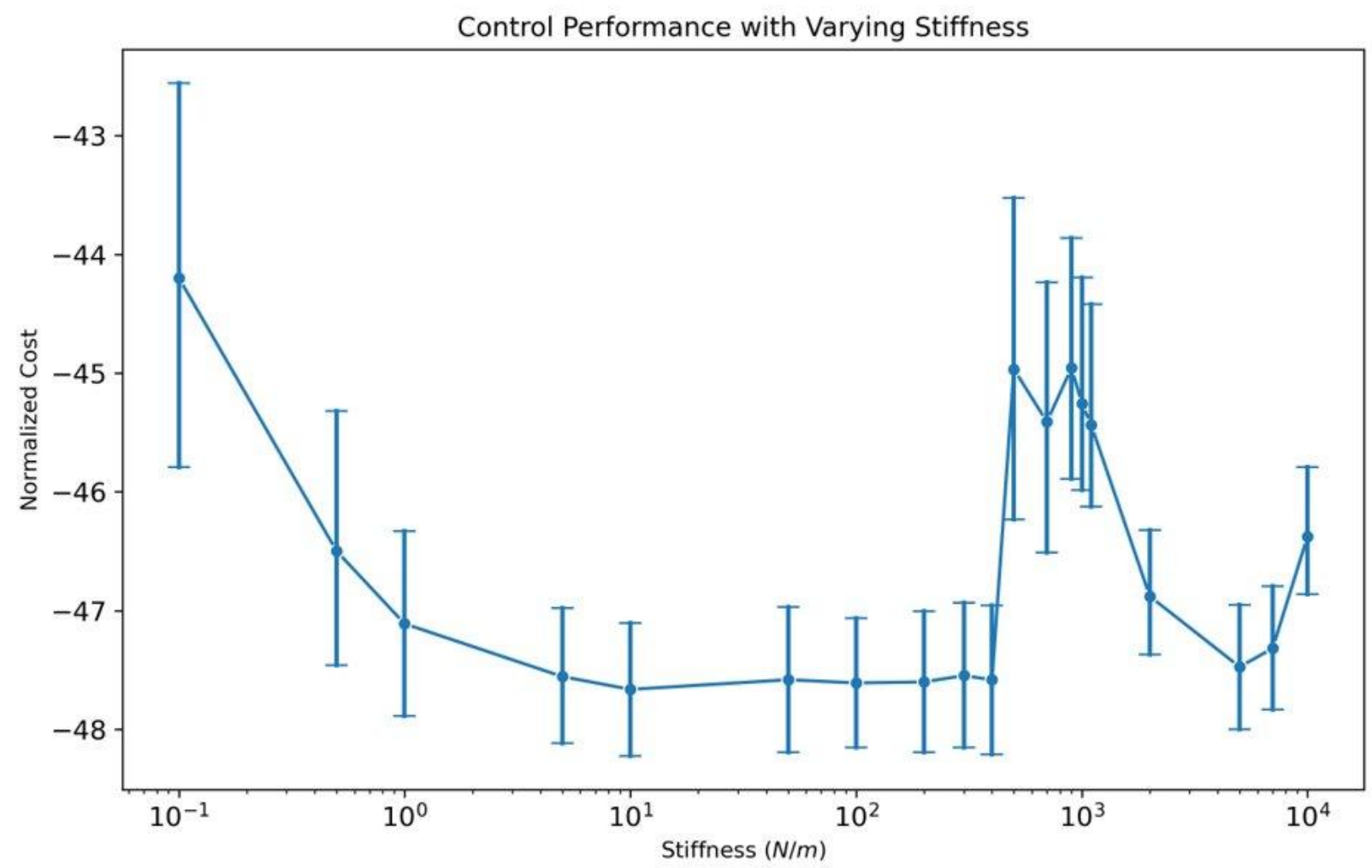

Supplementary Figure 1. Impact of Varying Pusher Stiffness on Square Slider Control Performance Varying the contact compliance in the pusher-slider system leads to changes in the performance of the controller. Very low and very high compliances perform the worst, with best performance occurring in the vicinity of stiffnesses on the order of 100N/m. The error bars represent the range of performance recorded from 5 different initial positions while tracking a constant-velocity trajectory in the positive x direction.

These numerical simulations validate that CCK-MPC performs well at pusher stiffnesses on the order of 100N/m, but loses performance in significantly higher or lower stiffness regimes (Supplementary Fig. 1). These results imply that there may be competing factors at low and high stiffnesses that impact the performance of CCK-MPC. We hypothesize that these factors may be reduced predictive accuracy from Koopman models at higher stiffnesses, and reduced responsiveness to control inputs at lower stiffnesses. A more detailed exploration of this trend, and an evaluation of the hypothesized factors are presented in separate work[3].

Supplementary Discussion 1.3: Koopman Modeling of a Cart-Pole with Walls: A Canonical Example

To show the ability for Koopman models to control contact-based systems, we generate a simple Koopman LQR controller for the cart-pole system with compliant walls. This model, described in Aydinoglu et al.[4], is given by a simple cart-pole with two generalized coordinates ($\boldsymbol{\theta} = [x_c, \theta_p]$) and compliant walls that are centered a distance d from the origin. Here, the contact dynamics are a simple linear spring, whose contact force is given by:

$$0 \leq \lambda_1 \perp l \sin(\theta_p) - x_c + \frac{1}{k} \lambda_1 + d \geq 0 \tag{10}$$

$$0 \leq \lambda_2 \perp x_c - l \sin(\theta_p) + \frac{1}{k} \lambda_1 + d \geq 0 \tag{11}$$

where λ_1 and λ_2 correspond to the right and left wall reaction forces respectively.

We make the assumption that the angle deviation from the unstable equilibrium is small, so the control input is linear affine in the equations of motion. Thus, we are able to obtain the Koopman **B** matrix analytically from the equations of motion. This assumption works well with small wall distance d. However, a Koopman model can be constructed that takes into account the state dependent nature of the control input using the Control Coherent Koopman method, either using the actuator pre-filter method or modeling the actuator dynamics.

The equations of motion for the cart-pole system with compliant walls is given below:

$$\ddot{\boldsymbol{\theta}} = \mathbf{H}^{-1}(\boldsymbol{\theta})\left[\mathbf{C}(\boldsymbol{\theta}, \dot{\boldsymbol{\theta}}) + \mathbf{G}(\boldsymbol{\theta}) + \mathbf{u}\right] + \mathbf{J}_c(\boldsymbol{\theta})\,\boldsymbol{\Lambda} \tag{12}$$

$$\mathbf{H}(\boldsymbol{\theta}) = \begin{bmatrix} m_c + m_p & -l\, m_p \cos(\theta_p) \\ -l m_p \cos(\theta_p) & l^2 m_p \end{bmatrix} \tag{13}$$

$$\mathbf{C}(\boldsymbol{\theta}, \dot{\boldsymbol{\theta}}) = \begin{bmatrix} \dot{\theta}_p^2\, l m_p \sin(\theta_p) \\ 0 \end{bmatrix} \tag{14}$$

$$\mathbf{G}(\boldsymbol{\theta}) = \begin{bmatrix} 0 \\ -g\, l m_p \sin(\theta_p) \end{bmatrix} \tag{15}$$

$$\mathbf{u} = \begin{bmatrix} u \\ 0 \end{bmatrix} \tag{16}$$

$$\mathbf{J}_C(\boldsymbol{\theta})\boldsymbol{\Lambda} = \begin{bmatrix} 1 & -l\cos(\theta_p) \\ 0 & -l\sin(\theta_p) \end{bmatrix} \begin{bmatrix} -\lambda_1 + \lambda_2 \\ 0 \end{bmatrix} \tag{17}$$

To obtain an analytical B matrix for the Koopman model, we linearize the control input term:

$$\widetilde{\mathbf{B}}\, u = \begin{bmatrix} \dfrac{1}{m_c} \\ \dfrac{1}{l\, m_c} \end{bmatrix} u \tag{18}$$

No embedding compensation term was used for this Koopman model. The data was lifted with 25 radial basis function observables placed with K-means++ and the **A** matrix generated via least squares. The simulation and Koopman time step were both 0.01 seconds, and the dynamics were integrated using forward Euler.

In Supplementary Figure 2, we show the results of the Koopman LQR model compared to a typical cart-pole LQR controller with no knowledge of the walls. The same state and control weights were used between the two controllers. The regular LQR controller rebounds against the walls and is unable to stabilize, while the Koopman LQR controller hits the right wall but is able to bring the cartpole to the origin. For 1000, six-second simulations, 79.9% of the simulations

using the KLQR controller had an average MSE in the last second below 0.01, but only 10.7% of simulations below 0.001. Contrast this with the regular LQR controller, who for the same initial conditions was able to get 43.7% of simulations below 0.01 and 42.8% below 0.001. While the Koopman LQR controller was more consistent in stabilizing the cart-pole, small errors in the model keeps the cart-pole from arriving at the unstable equilibrium. Regular LQR can bring the cart-pole to this unstable equilibrium, but only if the model is not greatly impacted by the wall collisions. The initial conditions for these simulations were uniformly sampled from the following ranges:

$$|x_c| \leq d, |\dot{x_c}| \leq 1.5 \tag{19}$$

$$\theta_p = 0, |\dot{\theta_p}| \leq 1 \tag{20}$$

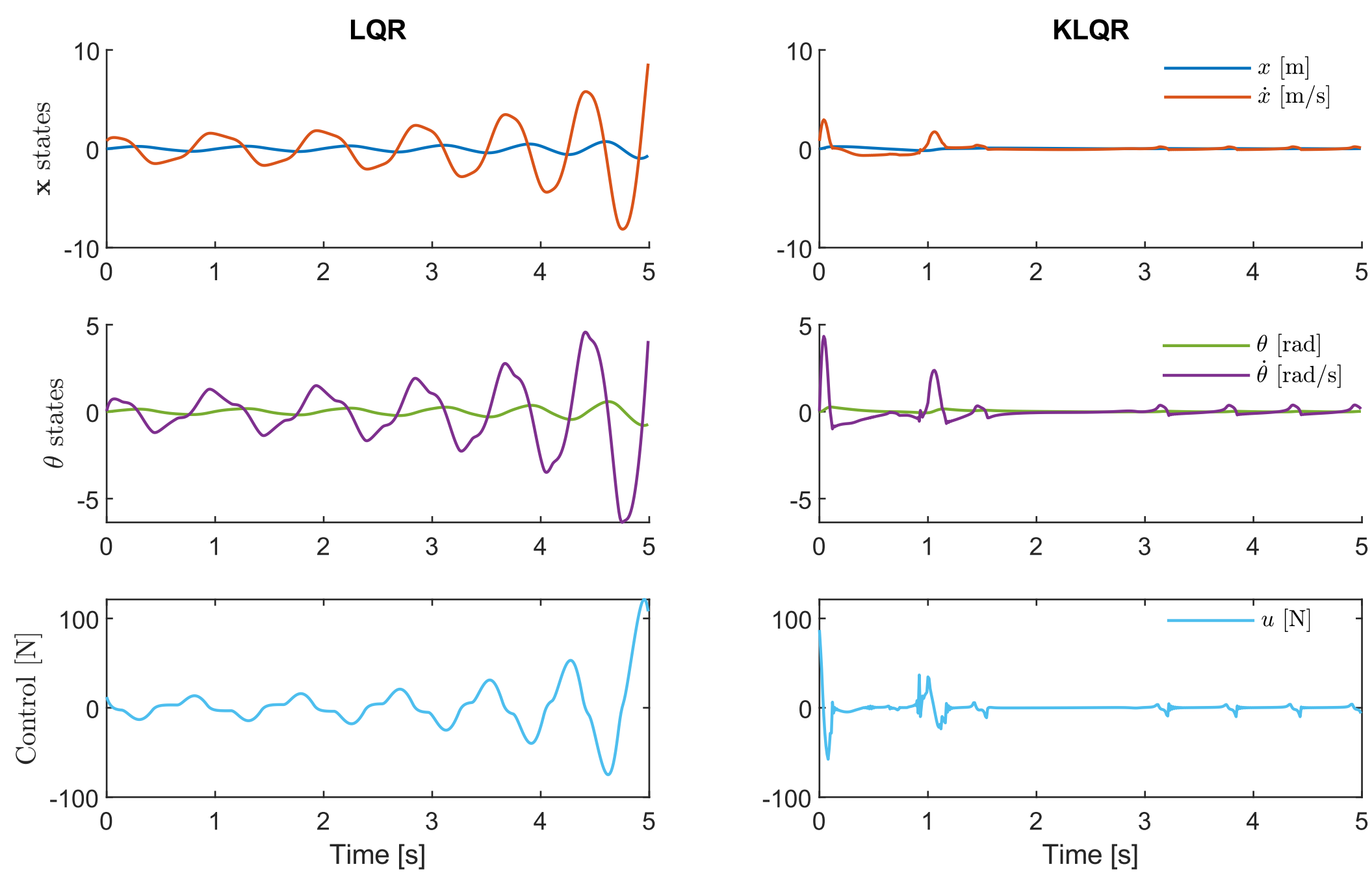

Supplementary Figure 2. Comparison of Koopman LQR vs LQR for Cart-Pole with Walls
Shown for a single trajectory. KLQR, which has knowledge of the wall location and dynamics, is capable of stabilizing the cart-pole, while the LQR controller bounces off the walls repeatedly.

Supplementary Discussion 1.4: Additional Comparisons of CCK-MPC to Bilinear Koopman MPC and L-MPC Approaches

In this section, we compare the performance of CCK-MPC to Bilinear Koopman (Supplementary Fig. 3), Linear MPC, and Time-Varying Linear MPC approaches (Supplementary Fig. 4). The dynamic planar pushing example is used for the purpose of this comparison.

Bilinear Koopman methods have emerged as a new tool for constructing data-driven Koopman models for dynamic systems. These approaches relax the purely linear constraint of the original

Koopman theory to accept bilinear models of the form $\mathbf{x}_{t+1} = \mathbf{A}\mathbf{x}_t + \mathbf{B}(\mathbf{x}_t)\mathbf{u}_t$, in which the control-input matrix $\mathbf{B}(\mathbf{x}_t)$ is a function of the system's state. To compare the performance of a bilinear Koopman model with the CCK approach described in this work, a bilinear model was trained on the circle slider's dynamics. The structure of the neural net was kept similar to that used for the CCK model, except with the addition of a state-dependent $\mathbf{B}$ component. To accommodate this, the training data was augmented to include randomized control inputs sampled from a uniform distribution of $\mathbf{u}_1 = [-0.01, 0.01]$ and $\mathbf{u}_2 = [-0.5, 0.5]$. This naturally leads to a larger amount of training data being required for the construction of the bilinear model, along with a corresponding increase in training time.

As is shown in the associated supplementary figure, the bilinear model underperformed when compared to the CCK approach in terms of position squared error over the course of a controlled trajectory. However, we wish to emphasize that this result does not indicate that CCK will always outperform a bilinear method. Both techniques are data-driven and are dependent on several hyperparameters. Given that bilinear models are more expressive than one which is constrained to a linear structure, it is reasonable to assume that more fine tuning, and even more training data and time would benefit improve the bilinear approach. However, the increased computation time that results from a bilinear structure is a more fundamental challenge. Explicitly using a bilinear model for the dynamics results in a non-convex optimization for MPC, which we solved using the Gurobi solver. While it may be possible to improve the computational efficiency of the bilinear KMPC method through careful construction of the optimization problem, the advantages of using a purely linear structure to enable convex MPC can be seen in the nearly two orders of magnitude improvement in the computational time of CCK-MPC when compared to bilinear KMPC.

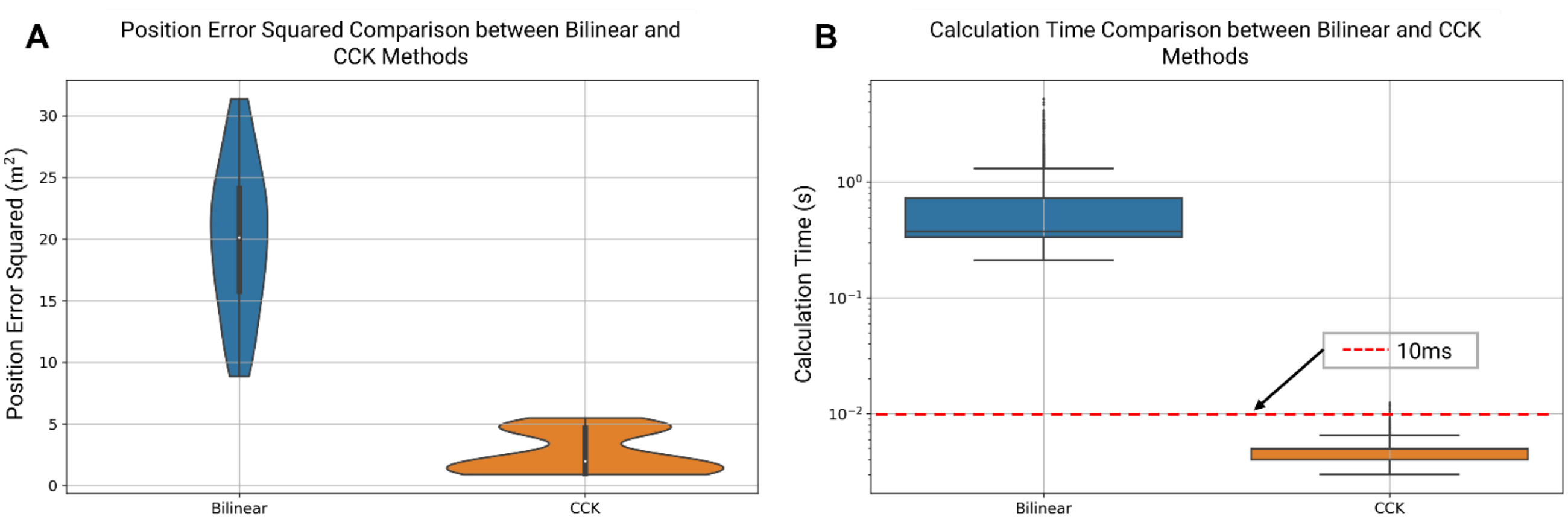

Supplementary Figure 3. Results from Comparing CCK-MPC with a Bilinear Koopman MPC Method
Numerical experiments using the circle-slider pusher system were performed that sought to move the slider to a fixed goal position. Trajectories were generated for 48 initial conditions. (**A**) the sum of the position squared error across each individual trajectory are plotted and compared. While the bilinear Koopman method does move the slider towards the goal, it is outperformed by the CCK implementation. (**B**) the bilinear structure used in bilinear KMPC results in a non-convex optimization problem. This leads to dramatically increased computation times of approximately two orders of magnitude when compared to the CCK method described in this work.

We have also compared the performance of KMPC to Time Varying Linear MPC (TVLMPC) and Linear MPC (LMPC) when tasked with pushing the circular slider towards a fixed goal. This comparison was performed across a set of 45 initial conditions for each of the linearization approaches. This additional comparison is presented below.

In our implementation, we made the following distinction between TVLMPC and LMPC:

- LMPC – a linearization is created from the initial state of the pusher-slider system and used throughout the task.
- TVLMPC – at each time step, the current state of the pusher-slider system is used to perform a local linearization. This ensures that the local linear model is as accurate as possible within each discretized time step. However, the system accumulates errors due to its inability to plan across mode boundaries, since the linearized A matrix is kept constant during each iteration of MPC although the predicted state goes beyond the boundaries.

Our results indicate that KMPC can outperform both local linearization methods in terms of control performance. This is especially the case with regards to the tail-end of the distribution of our test set, where contact state must be changed. See Supplementary Figure 4A below. KMPC's sum of squared error is an order-of-magnitude smaller than its counterparts.

In terms of computation time, we unsurprisingly find that LMPC is the fastest of the linear methods. While TVLMPC is the slowest due to the need to recompute the linear matrix in each time step, it is possible that the use of a dedicated automatic differentiation library could improve its computational speed. It is also possible that there is a more efficient way to structure the convex optimization problem with varying **A** and **B** matrices than what we used in Gurobi.

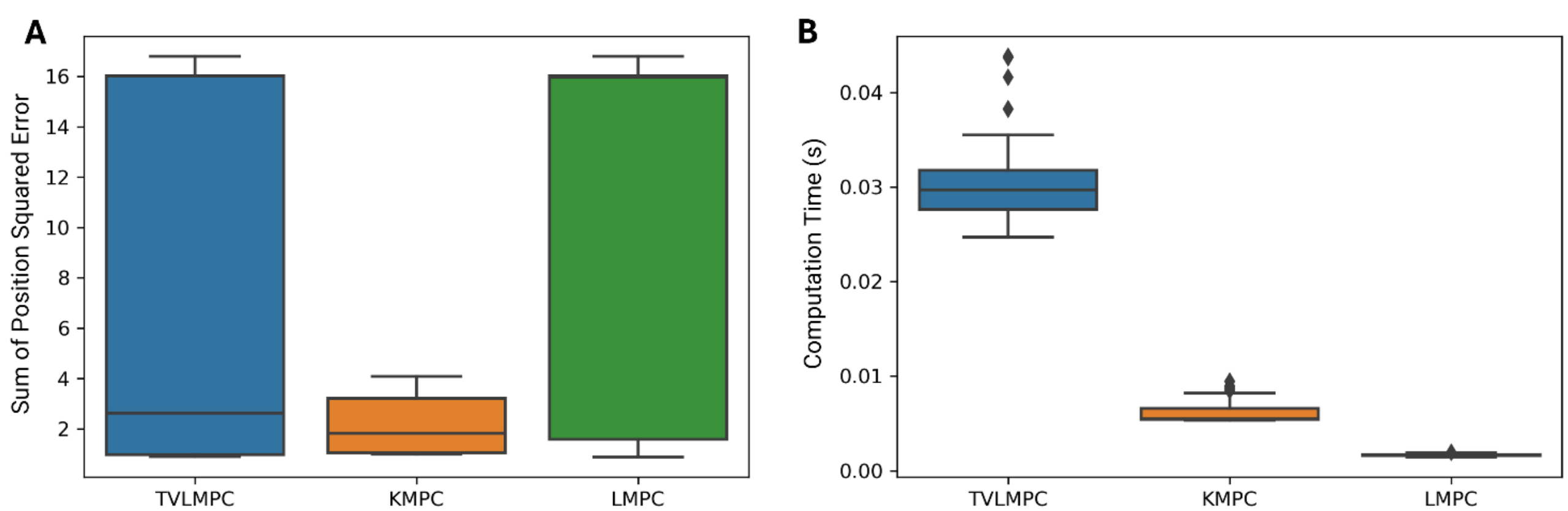

Supplementary Figure 4. Comparison between KMPC and Local Linearization Methods for the Circular Slider. The KMPC method presented in this work is compared against two alternative linear methods: Time Varying Linear MPC (TVLMPC) and Linear MPC (LMPC). (**A**) KMPC outperforms TVLMPC and LMPC in terms of control performance for the given task. This is especially the case in the tail end of the test distribution, with both TVLMPC and LMPC underperforming with more challenging initial conditions. (**B**) A key benefit of LMPC with a constant linear model is the fast computation enabled during convex optimization. Nevertheless, KMPC is still able to achieve sufficiently fast times of under 10ms.

Given this, we would caution against drawing too strong of a conclusion with regards to the comparative speed of KMPC and TVLMPC from our results.

Supplementary Discussion 1.5: Effect of Ablation of Koopman Observables

Koopman models can be heavily impacted by the quality of the nonlinear functions being used to represent the embedding space, as well as by the overall number of observables being used. When a DKN is used to approximate a Koopman – as in the pusher-slider examples in this work – the qualitative structures of the observables are learned from data. This leaves the number of observables as the primary parameter for tuning the quality of the final linear model. In general, a greater number of observables is expected to produce more accurate linearization, although data limitations introduce the possibility of over-fitting.

Different numbers of observables were considered while preparing the models used in this work. While there was indeed a general trend of improved performance with larger numbers of observables, we did not observe significant improvements for the pusher-slider system. It is possible that more training data may improve the larger models' performance, or that increasing the model size further may produce further gains. However, larger models also come at a non-negligible cost to the computational performance of the convex optimization problem. As such, we ultimately chose to focus on models with 100 observables in the main body of this work.

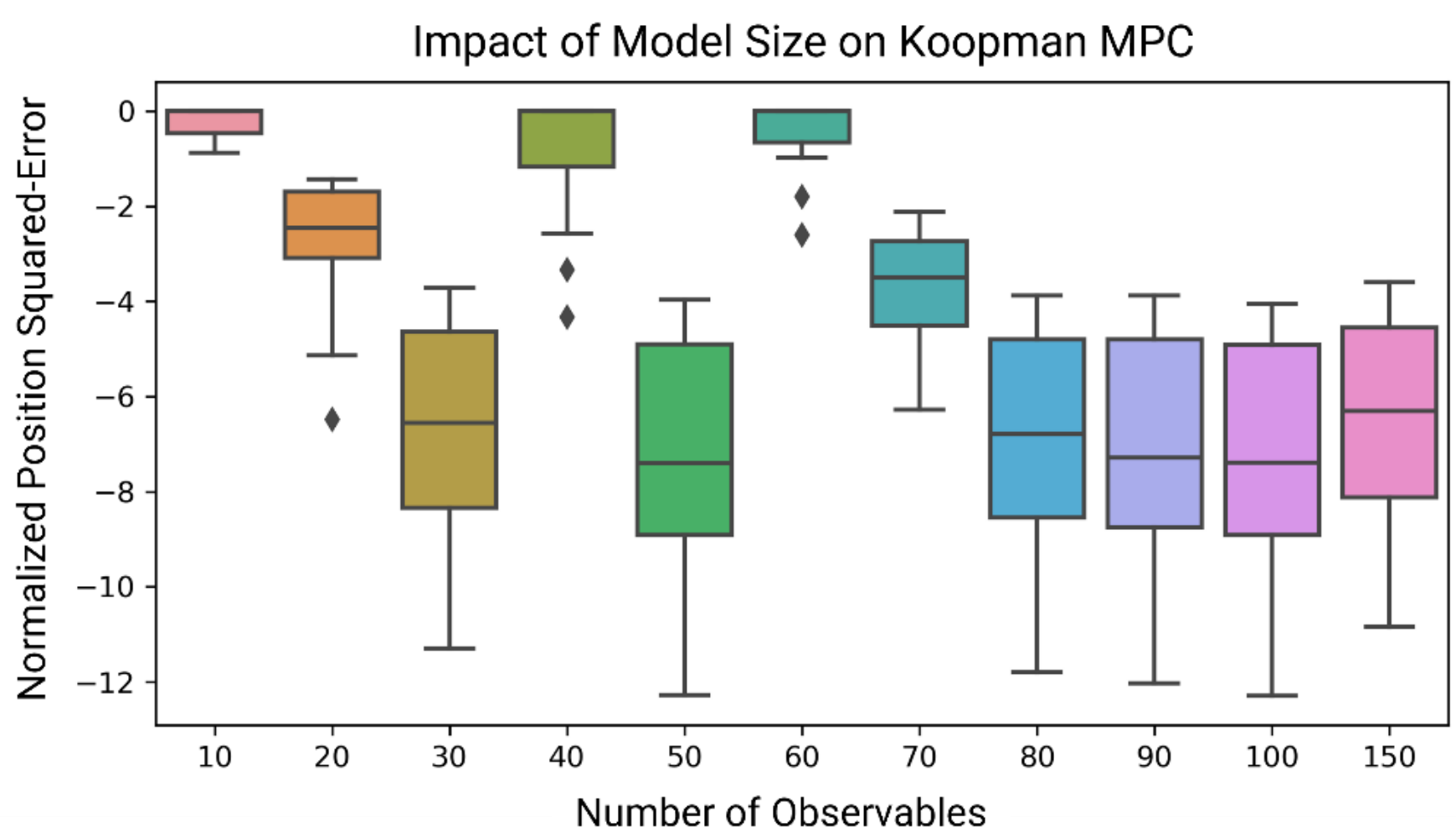

Supplementary Figure 5. Impact of Model Size on Koopman MPC
The circle-slider pusher system was tested with varying numbers of observables. Only a minor trend in improved performance is observed as the size of the models is increased. Note that the x-axis is not to scale. For each number of observables, one model was created and tested on 16 trajectories with different initial conditions. The sum of the squared position error for each of these trajectories was normalized by subtracting the cost of a no-control policy. As such, a negative value indicates better performance when compared to the no-control policy, while positive values indicate that the controller performs worse than when no control is applied at all.

Supplementary Methods

Supplementary Method 2.1: Computation of $\mathbf{A}$ and $\mathbf{B}$ matrices for the Rimless Wheel

The CCK $\mathbf{B}_p$ matrix was analytically derived from the linear relation between the control input and the actuator dynamics. From the equations of motion of the actuator dynamics,

$$I_m \ddot{\psi}_i = \left(\frac{\partial \mathbf{r}_i}{\partial \psi_i}\right)^T \cdot \mathbf{F}_i + u_i, \tag{21}$$

and with forward integration, the control input only affects actuator velocity in a current time step. Furthermore, since each linear actuator has a fixed inertia, the control input is brought in linearly and with a constant coefficient to the actuator states. The Koopman $\mathbf{B}$ matrix has the following form:

$$\mathbf{B}_p = \begin{bmatrix} \frac{\Delta_k}{I_m} & 0 & 0 \\ 0 & \frac{\Delta_k}{I_m} & 0 \\ 0 & 0 & \frac{\Delta_k}{I_m} \end{bmatrix} \tag{22}$$

$$\mathbf{B} = \begin{bmatrix} \mathbf{0}_{9\times3} \\ \mathbf{B}_p \\ \mathbf{G}\mathbf{B}_p \end{bmatrix} \tag{23}$$

Note that the $\mathbf{B}_p$ matrix has components only for three actuators. Motivated by the curse of dimensionality, the Koopman model employed in this paper models only the bottom three spokes as actuated. The rimless wheel is symmetric, with each spoke having similar dynamics. Furthermore, only one side of two aligned spokes can be in contact with the floor. This allows us to reduce "active" spokes from six to three, and the number of state variables from 18 to 12.

To generate the system matrix $\mathbf{A}$ of the associated autonomous system, physically relevant data was collected from simulated trajectories over $T_f = 2$ seconds initialized within a defined range. The trajectory was initialized with the actuated spokes as the bottommost spokes, that is $\theta \in [-\frac{\pi}{3}, \frac{\pi}{3}]$. To keep the simulation from failing, a stabilizing control input with small feedback gains was fed to the spokes in contact with the ground to counteract the ground reaction forces. With an equal simulation time step and sampling time step, combined with the CCK actuator modeling formulation, the control input can be added or removed from the non-lifted states such that

$$\mathbf{x}_{t+1}^{\text{passive}} = \mathbf{x}_{t+1}^{\text{actuated}} - \begin{bmatrix} \mathbf{0}_{9\times3} \\ \mathbf{B}_p \end{bmatrix} \mathbf{u} \tag{24}$$

where $\mathbf{B}_p$ is the constant matrix given in Supplementary Equation (22). The trajectory was prematurely ended if the inactive side of the spokes contacted the ground or if the reaction forces on the ground were excessively large.

The *passive* datasets were formulated from single time step pairs $\mathbf{x}_t$ and $\mathbf{x}_{t+1}^{\text{passsive}}$ for 2000 trajectories using the three-actuator model. The data was normalized with respect to characteristic lengths and velocities inherent to the system to alleviate artificial weighting due to dimension. 100 radial basis functions (RBF), chosen for their ability to encode spatial information, were placed on the normalized dataset, and the datasets were lifted to produce $\mathbf{z}_t$ and $\mathbf{z}_{t+1}$. The RBF's dilation factors were tuned to minimize the MSE of a validation dataset. Finally, the Koopman **A** matrix was generated from the lifted data using the least squares method.

To generate the DMDc model, both the passive dataset described above and the actuated dataset (with the control input included) were used. Data was concatenated with the corresponding control inputs and then lifted using RBFs defined solely as functions of the state. Using this lifted dataset, the **A** and **B** matrices were computed via least squares. The MSE was found to be lowest for very wide RBFs, the resulting **A** and **B** matrices were numerically ill-conditioned. Instead, a dilation factor corresponding to a local minimum of the MSE was selected, providing better numerical conditioning.

We also compare the prediction and control performance of the CCK model with and without the embedding compensation terms. As described in the Methods section of the main text, the embedding compensation term updates the embedding observables g. In Supplementary Equation (23), the term $\mathbf{GB}_{\boldsymbol{p}}$ given by $\mathbf{A}_{gp}\mathbf{A}_{\boldsymbol{pp}}^{-\mathbf{1}}\mathbf{B}_p$ was used for updating the embedding observables given a control input. The Koopman model that does not have the embedding compensation term, has a Koopman **B** matrix of the following form:

$$\mathbf{B} = \begin{bmatrix} \mathbf{0}_{9\times3} \\ \mathbf{B}_p \\ \mathbf{0}_{100\times3} \end{bmatrix} \tag{25}$$

By default, the compensation term is used unless stated otherwise.

Note that in the creation of the RBFs and Koopman models, the horizontal hub position x information is omitted. x does not appear in the dynamic equations of this system, and supplying the Koopman model with this uninformative data can lead to the learning of an x-dependency.

Supplementary Method 2.2: Rimless Wheel MPC Formulation

With the creation of the Koopman model, we define the MPC problem as shown below:

$$\min_{\mathbf{u}_0,\dots,\mathbf{u}_{N-1}} \sum_{t=0}^{N-1} (\tilde{\mathbf{z}}_t^T \mathbf{Q} \tilde{\mathbf{z}}_t + \mathbf{u}_t^T \mathbf{R} \mathbf{u}_\mathrm{t}) + \tilde{\mathbf{z}}_N^T \mathbf{Q} \tilde{\mathbf{z}}_N \tag{26}$$

$$\mathbf{u}_{t,\min} \leq \mathbf{u}_t \leq \mathbf{u}_{t,\max}$$

where the control input and actuator states are bounded. Note the positional error is calculated in the lifted space. Depending on the choice of observables, the observable error may be informative to achieve a desired behavior. In our implementation, the $\mathbf{Q}$ matrix values associated with observable error are relatively very small.

The MPC is called at the same rate as the Koopman time step ($\Delta_k = 0.01$ seconds) and solved using the Gurobi Optimizer. The control is implemented in simulation with a small fixed time step ($\Delta_s = 0.001$ seconds), so the control input is implemented as a zero-order hold. In this work, the delay due to computation is not considered. When MPC is called, the current state is nonlinearly mapped to the three-actuator model, normalized, and lifted. For more details on the implementation, see the pseudocode in Supplementary Fig. 6. The nonlinear mapping occurs once per function call and is performed outside of the linear solver.

To achieve forward rolling, a desired trajectory is obtained from the rimless wheel rolling passively down a ramp. This reference trajectory is faster than the actuated rimless wheel can achieve on flat ground because of the steep ramp angle ($20°$). It was found that simply setting the reference trajectory to be a stationary point far in the x-direction was insufficient in getting the wheel to roll successfully. The segment of the desired trajectory to track for a given MPC iteration is obtained by finding the closest match in terms of angle and angular velocity, defined in the least squares sense.

The $\mathbf{Q}$ matrix primarily weights error in x position and x velocity, with a slight penalty on y to keep the rimless wheel close to the floor. There is additionally a relatively small penalty to observable errors. No other non-lifted states are weighted, so the rimless wheel must determine the correct actuation sequence with minimal guidance.

Once Gurobi outputs an optimal control sequence, the control signal corresponding to the first time step is denormalized and mapped to the correct spokes. To keep the upper spokes entirely stationary, a control law is implemented to bring $\dot{\psi}_{\text{upper}} = 0$. The control effort to keep the top spokes stationary does not affect the wheel's rolling. To account for model inaccuracies, a separate controller ensures the actuator states (rotor angle and angular velocity) remain within predefined bounds. If the model fails to accurately predict ground reaction forces, the actuator may over-accelerate, leading to an infeasible state according to the constraints set in the MPC formulation.

When using local linearization in MPC with large time horizons, Gurobi often returns infeasible solutions. This likely occurs because the linearizations become increasingly inaccurate and potentially unstable as the time horizon increases and the states deviate further from the point of linearization. To enable the solver to return a control command in these cases, the constraints are relaxed, reformulating the objective to minimize the sum of the squared constraint and bound violations. However, local linearization with a shorter time horizon does not require constraint relaxations. While Fig. 4 and Supplementary Movie 3 show results for LL with N = 20 (where relaxed constraints were used), the same trials were also conducted with N = 2 and no constraint relaxation, with little to no improvement. The average and standard deviations across all trials are reported in Supplementary Table 5.

Supplementary Method 2.3: Additional Information for Implementing CCK-MPC on the Planar Pusher

We present some extra details regarding our implementation of CCK-MPC for the planar pusher that may be helpful for reproducing the results. Specifically, we highlight the use of additional linear constraints in the optimization to better bound the problem. A brief discussion on the need to “reset” the local reference frame of the circle slider is also presented.

2.3.1 Square Slider

As is standard, the dynamics of the system are incorporated into the constraints of the optimization problem. Although the linear Koopman model incorporates the dynamics for the states $\theta, p_x, p_y, \dot{x}, \dot{y}, \dot{\theta}$, the position of the slider must be determined separately. This is done through the trapezoidal integration of the slider velocities, as approximated using the Koopman model. While this approach would lead to exact estimates for a truly discrete system, the continuous nature of the real planar pusher problem means that we accumulate some positional error over time. Fortunately, this can be mitigated by taking smaller time steps, improving the accuracy of the Koopman model, and restricting CCK-MPC to a reasonable time horizon. In practice, we found that the improved computational performance from the reduced-order models outweighed the downsides of approximating the slider’s positional states in this way.

In addition to the dynamics of the system, the optimization program’s linear constraints also allow us to restrict the behavior of the controller to certain bounds. The square pusher had two such constraints:

- a set of constraints that prevent the pusher from moving beyond the boundaries of the slider’s edge. That is, p_y is constrained by the length of the slider, L, such that $|p_y| < \frac{0.91}{2}L$. This is necessary since learning the discontinuity in the dynamics of the pusher-slider system at the end of the slider’s edge is beyond the scope of this paper. While it is feasible that a Koopman model may be able to encapsulate these more complex dynamics, we specifically focused on the scenario of the pusher only contacting a single edge of the slider.

- a set of constraints that restrict the angular rotation of the slider such that $|\theta| \leq 40°$. This constraint is relaxed for the final step in the time horizon during the predictive loop, such that $|\theta| \leq 80°$. This constraint helps to prevent CCK-MPC from performing especially aggressive control inputs that induce large rotations in the slider.

2.3.2 Circle Slider

As with the square slider, we observed that the positions x and y were not necessary to predict the system’s dynamics. As such, they were not considered by the DKN. Only one additional constraint was added to CCK-MPC. Its purpose was to restrict the distance between the pusher and the slider to restrict the behavior of the system and improve performance: $p_1 \leq 0.1$. Otherwise, the crucial difference between the square and circle slider implementations is the need to “reset" the circle’s local reference frame based on the current configuration.

The need for this reset is due to the presence of multiple, isolated fixed points in the dynamic system. As with the square slider, there is a manifold of configurations in which the system of

stable when the pusher is not in contact with the slider. However, we also encounter a disconnected fixed point that may exist when the pusher is located at the center of the circular slider. Any linear system - even in a higher dimensional lifted space - cannot have multiple, isolated fixed points.

According to Koopman theory, it is possible to find a set of observable functions which map such a multiple-fixed-point system to a lifted representation in which none of the fixed points are isolated. However, this is topologically impossible if we require that the original states are included in the list of observables.

This can be understood with a simple thought experiment: imagine a system with a pair of isolated fixed points. If we were able to represent this system in a lifted space that includes the original states, then for a linear representation to be valid there would need to be a continuous path of fixed points in the lifted space that join the two original equilibria. Such a path would necessarily cross other locations in the original state space, which is incorrect.

We overcome this issue by producing a Koopman model for a restricted set of $p_2 \in [-\frac{\pi}{4}, \frac{\pi}{4}]$. This pushes the DKN to learn the dynamics of a specific “slice" of the circle slider. Beyond this slice, the linear model is free to adopt whatever dynamics may be mathematically admissible - even if such dynamics are radically different to that of the actual circle slider system.

Such an approach is appropriate in our case since CCK-MPC operates over a finite time horizon. If we assume that the linearized system will not evolve beyond the “slice" within the time horizon, then we don’t require a high accuracy for the rest of the slider. Between CCK-MPC iterations, a coordinate transform can be performed such that the local reference frame of the circle is such that $p_2 = 0$ - effectively mapping the true state of the system so that it is aligned with the “slice" model linearized by our Koopman approximation.

Supplementary Figures

Algorithm 1 Pseudocode algorithm for the planar pushing implementation

```
Define: x_ref                          ▷ Define the desired positions of the slider.
z_ref ← φ(x_ref)                       ▷ Lift the desired slider positions
t ← 0
x ← x_0                                ▷ Measure the initial state
while t ≤ t_f do
    z ← φ(x_t)                         ▷ Lift the current state
    u ← CCK-MPC(z, z_ref)              ▷ Calculate the control input via CCK-MPC
    Apply: u                           ▷ Apply the control input
    Wait: Δt
    t ← t + 1
    x ← x_t                            ▷ Measure the current state
end while
```

Supplementary Figure 6. Pseudocode Algorithm for the Planar Pusher
A summary of the algorithm used by CCK-MPC during runtime, presented as a pseudocode diagram.

Supplementary Tables

Supplementary Table 1. Parameter values for the Rimless Wheel Dynamics.
Values estimated from the LINAK LA36 linear actuator. Control inputs never reached the maximum control bound, as doing so would result in accelerations that exceed the actuator's velocity limits.

Subsystem	Parameter	Symbol	Value	Dimension
Wheel Body	Central Mass	m	25.0	kg
	Spoke Length	l	0.425	m
	Wheel Inertia	I	1.45	$\mathrm{kg\,m^2}$
Ground Contact	Ground Stiffness	k_y	100	$\mathrm{N\,m^{-1}}$
	Ground Damping	b_y	5000	$\mathrm{N\,s\,m^{-1}}$
	Max Ground Penetration	l_{max}	0.1	m
	Friction Coefficient	μ	0.8	—
	Pseudo-Coulomb Parameter	λ	0.001	—
Actuator	Max Actuator Displacement	ψ_{max}	165	rad
	Max Actuator Velocity	$\dot{\psi}_{max}$	352	$\mathrm{rad\,s^{-1}}$
	Max Control Bound	u_{max}	560	N m
	Total Motor Inertia	I_m	0.0012	$\mathrm{k\,g\,m^2}$

Supplementary Table 2. Parameter values for the Rimless Wheel Koopman models and Simulation.
The $\boldsymbol{Q}$ matrix is zero except for the $x, \dot{x}$ and embedding observable diagonal elements. L-MPC parameters are the same for CCK, DMDc and LL models. The reported $\boldsymbol{Q}$ and $\boldsymbol{R}$ weights are for normalized values.

	Parameter	Symbol	Value	Dimension
Simulation	Time Step	Δ_s	0.001	s
	Total Duration	T_f	20.0	s
CCK Model	# Passive Data Points	N_p	1.69e5	—
	# Observables	n	100	—
	RBF Shape Parameter	ϵ	2.00	—
	Koopman Time Step	Δ_k	0.01	s
DMDc Model	# Passive Data Points	N_p	1.69e5	—
	# Actuated Data Points	N_A	1.69e5	—
	# Observables	n	100	—
	RBF Shape Parameter	ϵ	1.50	—
	Koopman Time Step	Δ_k	0.01	s
L-MPC	Time Horizon	N	20	steps
	x Error Weighting	Q_x	2.0	−
	$\dot{x}$ Error Weighting	$Q_{\dot{x}}$	20	—
	Observable Error Weighting	Q_g	0.01	—
	Control Weighting	R_u	0.0028	−

Supplementary Table 3. DKN Hyperparameters
γ is the time weight, T is the time horizon during training, α_{obs} is the weight on observable loss, and α_{state} is the weight on state loss.

Hyperparameter	Square Pusher	Circle Pusher
α_{obs}	1	100
α_{state}	3	1

Supplementary Table 4. CCK-MPC Parameters for Planar Pushing
$\boldsymbol{Q}$ is the state cost matrix, $\boldsymbol{R}$ is the control cost matrix, T is the time horizon in terms of time steps with $\Delta t = 0.1s$. diag(·) refers to a diagonal matrix with the provided values lying along the diagonal.

**Parameter**	**Square Slider (Steady Push)**	**Square Slider (Shove)**	**Circle Pusher**
**Q**	$\text{diag}(100,100,1,0,\cdots,0)$	$\text{diag}(100,100,1,0,\cdots,0)$	$\text{diag}(10,10,0,\cdots,0)$
**R**	$\text{diag}(0.0007,0.009)$	$\text{diag}(0.00009,0.009)$	$\text{diag}(1e-6,1e-9)$
N	3	3	3

Supplementary Table 5. Comparison of Linearization Methods for use in L-MPC
The data presented is for ten trials, where the set of initial conditions is the same between models. The initial conditions were

sampled 0.05 seconds apart from the reference trajectory. The simulations ran for twenty seconds with the L-MPC parameters outlined in Table S3. The local linearization model was tested for both N = 20 and N = 2 time horizons.

Model	N	Maximum *x*-Displacement [m]		Average Absolute Angular Velocity [rad/s]	
		Mean	SD	Mean	SD
CCK w/ C	20	15.476	0.100	1.966	0.013
CCK no C	20	15.375	0.130	1.953	0.019
DMDc	20	0.719	0.084	0.124	0.005
LL	20	1.574	1.200	0.230	0.121
	2	1.167	0.167	0.214	0.024

Supplementary References

1. Asada, H. H. Global, Unified Representation of Heterogenous Robot Dynamics Using Composition Operators: A Koopman Direct Encoding Method. *IEEE/ASME Transactions on Mechatronics* **28**, 2633–2644 (2023).
2. Yeung, E., Kundu, S. & Hodas, N. Learning Deep Neural Network Representations for Koopman Operators of Nonlinear Dynamical Systems. in *2019 American Control Conference (ACC)* 4832–4839 (2019). doi:10.23919/ACC.2019.8815339.
3. O'Neill, Cormac. Koopman Dynamic Modeling and Control for Robotic Systems Making and Breaking Contact. *Doctoral Dissertation in the Department of Mechanical Engineering, Massachusetts Institute of Technology* (2025).
4. Aydinoglu, A., Wei, A., Huang, W.-C. & Posa, M. Consensus Complementarity Control for Multicontact MPC. *IEEE Transactions on Robotics* **40**, 3879–3896 (2024).
