# Supplementary material for: Koopman global linearization of contact dynamics for robot locomotion and manipulation enables elaborate control
Source: Nat Commun. 2026 Jun 19;17:7749. doi: 10.1038/s41467-026-72485-7 (PMC13434789; doi:10.1038/s41467-026-72485-7)
Supplement: Supplementary file 3 [file 41467_2026_72485_MOESM2_ESM.pdf]

Description of Additional Supplementary Files

File Name: Supplementary Movie 1
Description: CCK-MPC discovers a method to initialize rolling.

File Name: Supplementary Movie 2
Description: CCK-MPC with varying time horizons.

File Name: Supplementary Movie 3
Description: Comparison of linearization techniques for L-MPC.

File Name: Supplementary Movie 4
Description: Square slider experiment shoving results.

File Name: Supplementary Movie 5
Description: Circle slider experimental pushing results.

File Name: Supplementary Movie 6
Description: Circle slider simulated results with friction mismatch.

File Name: Supplementary Movie 7
Description: LQR and Koopman LQR for a Cart-Pole with Walls.
